# Millimeter Wave Multi-Port Interferometric Radar Sensors: Evolution of Fabrication and Characterization Technologies

**DOI:** 10.3390/s20195477

**Published:** 2020-09-24

**Authors:** Serioja Ovidiu Tatu, Emilia Moldovan

**Affiliations:** Institut National de la Recherche Scientifique, Centre Energie-Matériaux et Télécommunications, INRS-EMT, Montreal, QC H5A 1K6, Canada; moldovan@emt.inrs.ca

**Keywords:** antenna array, integrated circuits, interferometry, millimeter wave, multi-port technology, radar measurements, quadrature down-conversion

## Abstract

Recent advances in millimeter wave technologies, both in component and system design, in line with important size and cost reductions, have opened up new applications in ultra-high-speed wireless communications, radar and imaging sensors. The paper presents the evolution of millimeter wave circuit and modules fabrication and characterization technologies in the past decades. Novel planar low-cost fabrication technologies have been successfully developed in this period. In combination with the standard rectangular wave-guide technology, these offer great opportunities for prototyping and testing of future millimeter wave transceivers or front-ends, which integrate antenna arrays, down-converters, modulators, amplifiers, etc., in a compact fixture. The paper uses, as a suggestive example, the evolution of the multi-port interferometric front-ends implementation from millimeter wave bulky components and systems to miniaturized and high-efficient ones. Circuit and system designs are carefully done to avoid (as much as possible) complicated calibration methods or difficult post-processing of baseband data. This requires an increased effort in design and fabrication, but it allows miniaturization, low-power consumption, while keeping very good overall performances. Useful and straightforward laboratory characterization techniques of circuits and systems are described in detail.

## 1. Introduction

Recent advances in fabrication techniques allow increased performance, cost reduction and miniaturization of microwave and millimeter wave transceivers for high-speed wireless communications, radar and imaging sensors. Wireless high-speed communications target data-rates of several Gb/s that can be achieved with a reduced complexity and number of symbols, due to the available bandwidths, and with the reuse of millimeter wave frequencies, due to high values of free space attenuation. In the radar sensors domain, function of targeted measurements (speed, range, high resolution displacement, gesture sensing, frequency of mechanical vibrations, imagery) the system architecture, measurement, calibration and signal processing techniques must be properly considered by the designer.

Today’s conventional millimeter wave designs with discrete components use single diodes or pairs of opposite diodes to down-convert the signal, requiring a relatively high-power for the millimeter-wave local oscillator (LO) of around 10–15 dBm. A non-conventional technique is the use of multi-ports in the front-end design. This interferometric approach is based on the comparison between the input unknown signal and the reference one from the LO. A considerable reduction of the power consumption (the requested LO power is reduced with more than 30 dBm), of the size and the cost of the receiver front-ends is therefore obtained.

A wide area of potential applications emerges, from more conventional to new areas, such as: automotive sensors (range and relative speed measurements, imagery for autonomous vehicles); imaging sensors for security and surveillance; radar sensors for precision industrial measurements in harsh environments, radar sensors for non-contact measurements of vital functions and imaging sensors for cancer detection in medicine, radar sensors for the rapid development of IoT, radar sensors and multi-sensor systems for human gesture recognition, human-computer interaction, smart homes and games, combined radar/communication systems allowing detection and data-rate transmission between vehicles, devices, etc.

Design and characterization of multi-port circuits and radar sensor prototypes in different fabrication technologies, operating over several frequency bands (60, 77, 86 and 94 GHz), and for various applications will be presented. The paper intends to demonstrate the advantages of the millimeter wave interferometry in system designs.

## 2. Multi-Port Interferometry History and Basic Operation Principle

The multi-port (six-port) circuit was introduced in the 1960s, and was used exclusively in the first three decades as a low-cost alternative to expensive vector network analyzers [1,2,3,4,5,6,7,8,9,10,11,12,13,14,15,16,17,18,19,20,21,22,23,24,25,26,27,28,29,30,31,32]. Even with today’s development of electronics, it can play a similar role, especially over millimeter wave frequencies where the price of equipment is prohibitive, or for special applications requiring very low-cost measurement equipment. The idea was initially proposed by Cohn and Weinhouse [1] to evaluate the phase of a microwave signal, and extended by Engen and Hoer [2,3,4,5,6] for accurate automated measurements of the complex reflection coefficient in microwave network analysis.

The six-port is a passive circuit, composed of several couplers connected with transmission lines. It has two RF inputs (reference and signal to be measured) and four baseband outputs. The evaluation of the reflection and transmission coefficients of a device under test are based on the measurement of the output signal power levels using diode-based power detectors. With the help of a graphical construction, based on the intersection of three circles, the value of the reflection coefficient is obtained with a very good accuracy after calibration. More details are given in Section 4.1.2.

The idea of using a multi-port circuit in direct conversion microwave receivers for radars [33] or wireless communications [34,35] was proposed for the first time at the Poly-Grames Research Centre of “École Polytechnique de Montreal” between 1994 to 1996. The first reported results were related to the narrow-band single-carrier demodulation of digital data. The multi-port architecture was very similar to the original approach, and a calibration process was always used to obtain useful baseband information. Since 2001, multi-port interferometer transceivers operating over microwave or millimeter wave frequencies for various applications from wireless communications to radars have been developed [36,37,38,39,40,41,42,43,44,45,46,47,48,49,50,51,52,53,54,55,56,57,58]. All those applications use a quasi-similar multi-port architecture, as described further. Multi-port interferometers are no longer built with commercial couplers connected by coaxial cables, as in the starting years. Complicated calibration procedures are therefore avoided due to a careful circuit design and fabrication procedure, for each frequency band and specific targeted application.

**Figure 1:**
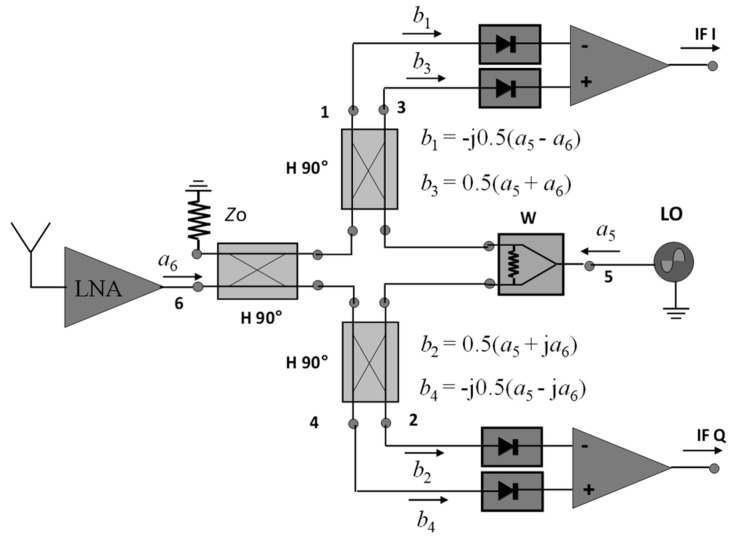
The multi-port interferometer down-converter in a front-end block diagram.

Figure 1 shows a typical implementation of a quadrature down-converter in a front-end block diagram [38]. The received signal from the antenna (or antenna array in mm-wave communication receivers or radars), amplified (if needed) by a low-noise amplifier (LNA), *a*_6_, is compared with the reference signal generated by the LO, namely *a_5_*. The multi-port, implemented with three quadrature hybrid couplers (H 90°) and a Wilkinson power divider (W) plays the role of an interferometer. The input signals at ports 5 and 6 are linearly combined as vectors and four output normalized power waves, *b*_1_ to *b*_4_ are obtained. After power detection, signals are amplified in a differential manner to generate quadrature signals IF I and IF Q with a minimum DC offset. The input signal *a*_6_ can have a frequency slightly different from the reference signal. In communication receivers this can carry modulated information (QAM, PSK) or can be simply a Doppler shift in the case of CW radar sensors.

The operating principle of the multi-port interferometer can be described in several equations:

a_5_ = a exp[ω_0_(t) + φ_5_(t)], (1)

a_6_ =α a exp[ω(t) + φ_6_(t)] = α a_5_ exp[∆ω(t) + ∆φ(t)], (2)

where:

∆ω(t) = ω(t) − ω_0_(t), (3)

∆φ(t) = φ_6_(t) − φ_5_(t), (4)

Supposing that identical power detectors, well matched, having a quadratic response, are connected to the six-port outputs, the detected output voltages are:

v_i_ = Kb_i_b_i_* (5)

It was previously demonstrated [38] that:

v_IF_^I^ (t)= A_IF_[v_3_(t) − v_1_(t)] = αKa^2^A_IF_ cos[∆ω(t) + φ(t)], (6)

v_IF_^Q^ (t)= A_IF_[v_4_(t) − v_2_(t)] = αKa^2^A_IF_ sin[∆ω(t) + φ(t)], (7)

where α is the ratio between normalized input waves (*a*_6_/*a_5_*) and *A_IF_* is the gain of the differential amplifiers.

According to the previous equations a quadrature down-conversion is performed. This is very important in radar sensors, where a straightforward relationship is obtained between the received signals in complex plane and the movement of the target. The vector turns clockwise or anticlockwise on a spiral, depending on the sense of movement of the target. The spiral trajectory is due to the fact that the received signal becomes stronger when the target is closer, and weaker when the target is further.

**Figure 2:**
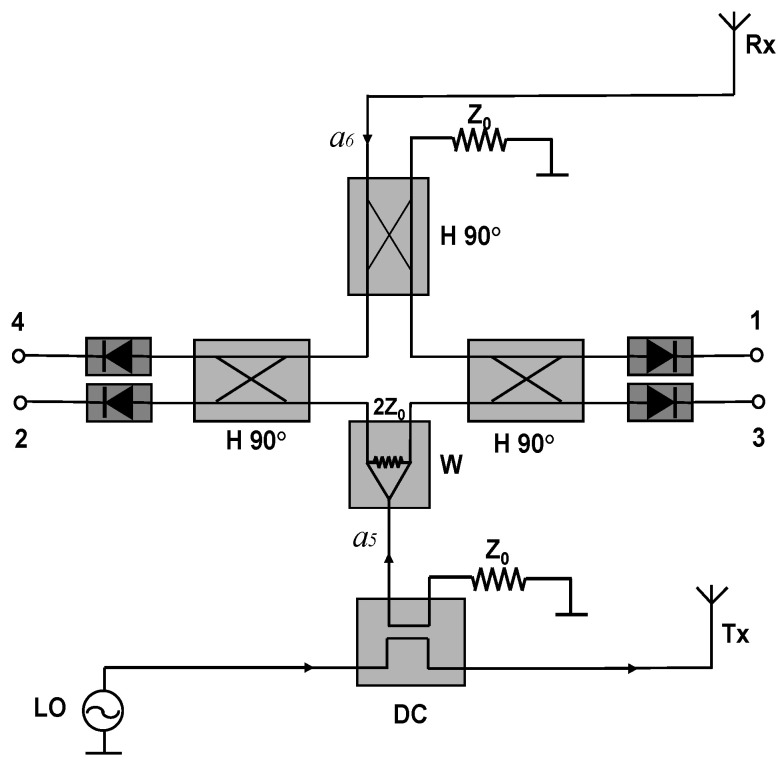
The multi-port interferometer bistatic radar sensor block diagram.

Figure 2 shows a typical implementation of a multi-port short-range bistatic radar sensor in which the reference signal at port 5 is obtained using a directional coupler (DC) from the transmitted signal [59]. If needed, a LNA can be connected between the receiving antenna and the port 6 of the multi-port. The quadrature differential output signals are generated at the odd and even outputs of the power detectors.

## 3. Fabrication Technologies

During the last decades, the millimeter wave fabrication technologies evolved from rectangular wave-guide (RWG), to planar technologies such as substrate integrated wave-guides (SIW), miniature hybrid microwave integrated circuits (MHMIC) and monolithic microwave integrated circuits (MMIC). Circuits have been miniaturized and costs have been dramatically reduced. The next sections will show some millimeter wave front-end implementations in the previously mentioned fabrication technologies. The multi-port architecture is very similar with the one presented in Figure 1 and Figure 2.

### 3.1. Wave-Guide Technologies

In order to validate the use of interferometric techniques for millimeter wave radars in the beginning years, a 94 GHz multi-port has been fabricated in conventional RWG in a brass block [59,60].

**Figure 3:**
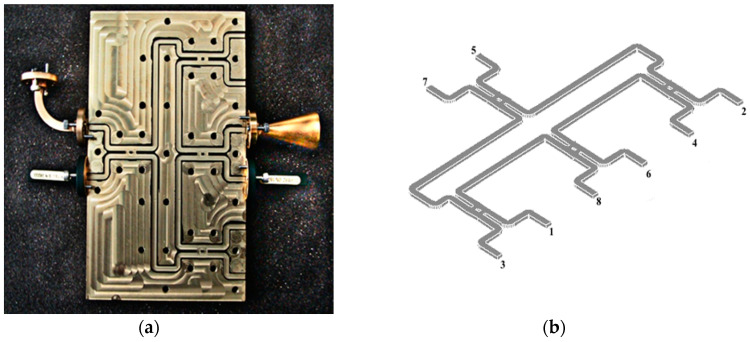
Layout of rectangular wave-guide (RWG) brass multi-port: (**a**) photo of a section; (**b**) lay-out.

Figure 3 shows a photo of a section of the circuit and its layout (115 × 78.5 mm). The guide dimensions correspond to the standard WR-10: 2.54 × 1.27 mm (100 L × 50 mil). The Wilkinson power divider has been replaced by a quadrature 90° hybrid coupler with a quarter wave section connected at one of the outputs.

This topology has been chosen because resistors cannot be integrated in RWG. The top of the multi-port is a simple metallic plate. The size and weight of the circuit are impressive (13 × 9 × 2.2 cm, about 2.25 Kg of brass). Standard WR-10 flanges are used to interconnect to laboratory equipment, horn antenna and matched loads (connected at the unused ports). The multi-port thickness is determined by the diameter of the standard WR-10 flange, equal to 19.05 mm.

**Figure 4:**
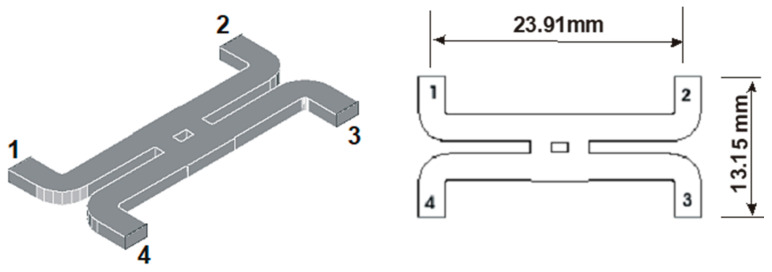
The layout of the 90° hybrid coupler fabricated in a brass block.

The main element of the multi-port is the 90° hybrid coupler. It was simulated using the High Frequency Structure Simulator (HFSS), a commercial finite element method solver for electromagnetic structures from Ansys. Figure 4 shows the layout and overall dimensions of the coupler.

**Figure 5:**
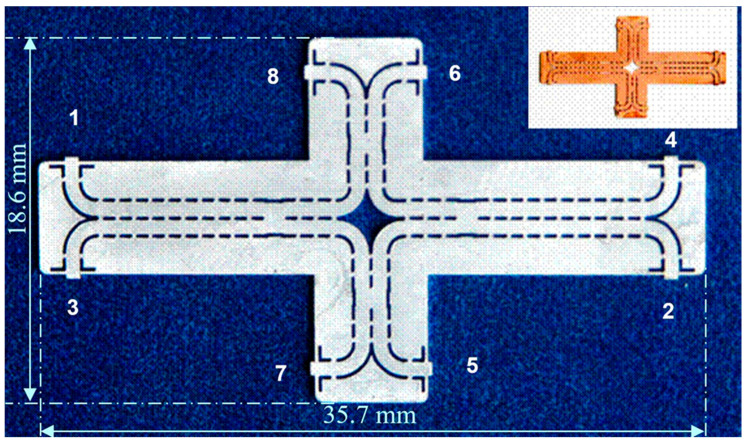
The layout of a substrate integrated wave-guide (SIW) multi-port fabricated in 10 mils ceramic.

A second step in development is the use of the SIW planar technology to reduce its size [61,62,63]. The SIW circuit has been fabricated on a 10 mils ceramic substrate (ε_r_ = 9.9). Figure 5 shows a picture of the circuit [64,65,66]. Its size is 35.7 × 18.6 × 0.254 mm. Unused ports (7,8) are connected to external matched loads. This architecture has also been chosen because resistances cannot be integrated in the SIW, like in the RWG technology.

**Figure 6:**
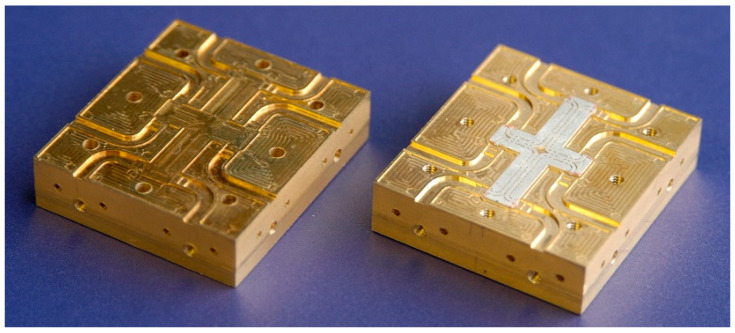
SIW multi-port in a brass fixture with standard WR-10 flanges.

In order to measure the circuit with conventional equipment the circuit was embedded in a metallic fixture, with WR-10 standard flanges, as seen in Figure 6. The cover top is flipped over the bottom and fixed with screws to obtain a compact module. As it can be seen, compared to the previous circuit, the conventional RWG is split into two parts, one in the top, and one in the bottom of the fixture. The bottom and the top brass pieces measure 43 × 50 × 11 mm each.

**Figure 7:**
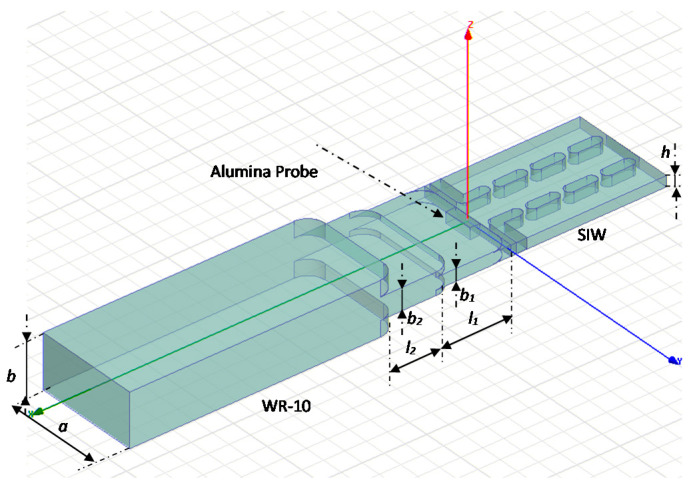
Details of the SIW standard WR-10 transition.

Figure 7 shows a detail of the SIW to WR-10 transition. The optimized dimensions of impedance transformers for 94 GHz are: *l*_1_ = 1.32 mm, *l*_2_ = 1.37 mm, *b*_1_ = 0.28 mm, *b*_2_ = 0.533 mm. In the same figure, *h* is the substrate thickness of 0.254 mm, and *a* and *b* are the standard dimensions of the WR-10 RWG, 2.54 and 1.27 mm, respectively. The alumina probe has the height of the dielectric substrate *h*, the width is equal to the SIW waveguide one and its length, optimized with HFSS is equal to 0.241 mm. Because of the mechanical characteristics of the milling machine, some corners of the structure have been rounded taking into account the minimum diameter of the head equal to 0.406 mm. The insertion loss of the transition is 0.1 dB at 94 GHz and less than 0.2 dB between 92 to 98 GHz. The return losses are 30 dB at 94 GHz and around 15 dB at the edges of the same band.

**Figure 8:**
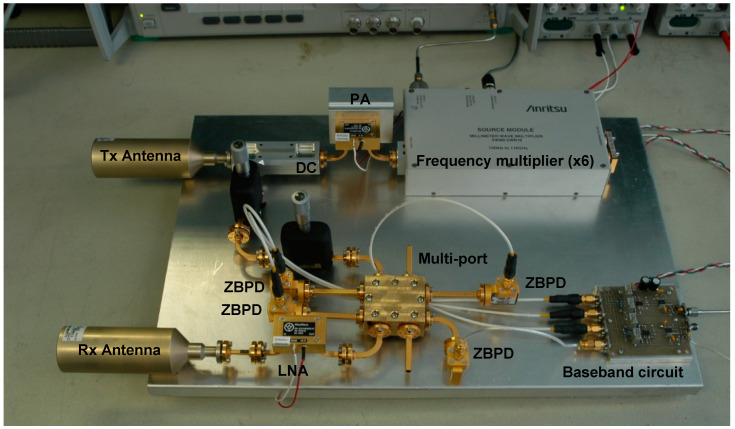
Photo of the 94 GHz radar sensors with WR-10 waveguide components and modules.

Figure 8 shows a W-band (94 GHz) radar sensor built with the SIW multi-port of Figure 6 [66]. Standard commercial waveguide components and modules are used for this initial radar prototype. The directive horn antennas type QRR-W00Y75 have a gain of 27 dBi, and a 3 dB beamwidth of 7.5°. The millimeter wave signal is obtained with an Anritsu frequency multiplier (6×) from a microwave source (15.66 GHz) and amplified with a 23 dB power amplifier (PA). The waveguide directional coupler of 10 dB in series with a 10 dB attenuator and a phase shifter are used for the reference signal of the multi-port. The LNA, connected to the receiving horn antenna has a gain of 33 dB. The wave-guide power detectors connected to the multi-port outputs are zero-bias type (ZBPD). The quadrature outputs are finally obtained with an in-house-built baseband circuit using OPA 2658 differential amplifiers.

### 3.2. Planar Integrated Technologies

In order to further miniaturize the multi-ports for radar sensors and integrate low-noise amplifiers, power detectors, antenna arrays, etc. in a single module, planar technologies must be used. Of course, in order to facilitate laboratory measurements, some connectors are necessary: RWG flanges for connections to laboratory frequency synthesizers (LO) and SMA connectors for quadrature outputs required to display signals on the oscilloscope screen, for example.

**Figure 9:**
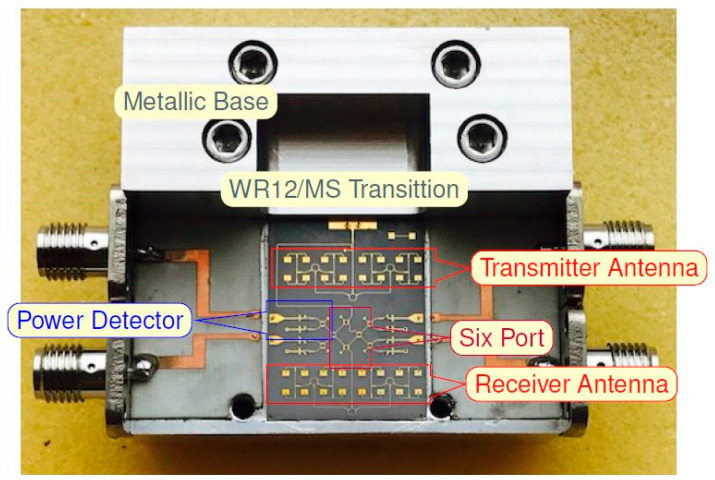
E-band multi-port radar sensor in aluminum fixture with standard WR-12 flange and SMA connectors.

Figure 9 shows an E-band radar sensor front-end, according to the block diagram of Figure 2 [67,68,69]. It is mounted in an aluminum fixture with a WR-12 standard flange and four output SMA connectors. The multiport, power detectors and related components, including two antenna arrays are fabricated in a single MHMIC die to minimize interconnection losses. The in-house MHMIC process uses 5 mil (127 µm) ceramic dies, having a maximum size of 2.5 × 2.5 cm. The relative permittivity of ceramics is ε_r_ = 9.9. The microstrip lines and antenna elements are 1 µm thick metallized (gold on ceramics), and integrated resistors are implemented with TiO_2_ with a 100 Ω/square resistance. This means that whatever the square dimension would be, it has the same resistance. Resistor values are obtained by modifying the L/H ratio of the TiO_2_ rectangle.

In the center of the image the multi-port, composed by four 90° hybrid couplers and a semi-circular meander line playing the role of a 90° phase shifter at the central operating frequency, can be easily identified. As for the previous waveguide sensors, the Wilkinson power divider has been replaced with a 90° hybrid coupler connected to a quarter waveguide transmission line at one of the outputs (meander line), to play a similar function.

Power detectors are implemented with a 90° hybrid ring coupler and two Schottky diodes each, in order to offer a very good matching over a wide frequency band, as explained further. Therefore, the entire multi-port structure contains eight 90° hybrid couplers and eight Schottky diodes, with related integrated loads. In order to increase the gain, 16-element antenna arrays are integrated for both Tx and Rx. The incoming signal from the WR-12 RWG feeds the Tx antenna array. A part of this signal, obtained with the 10 dB directional coupler is the reference signal at port 5. The received signal with the Rx antenna array (bottom) is injected at port 6 of the interferometer.

**Figure 10:**
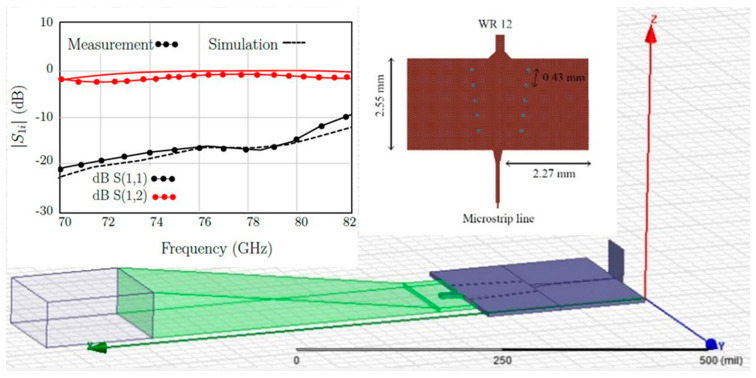
Microstrip to WR-12 transition: S-parameters and dimensions.

A novel microstrip to WR12 transition has been designed and optimized with the HFSS software [67]. The standard rectangular waveguide is WR-12: 3.1 × 1.55 mm (122 × 61 mils). The lay-out of this transition, simulation and measurement results are shown in Figure 10. This transition is mandatory to connect the planar circuits with the millimeter wave frequency synthesizer.

**Figure 11:**
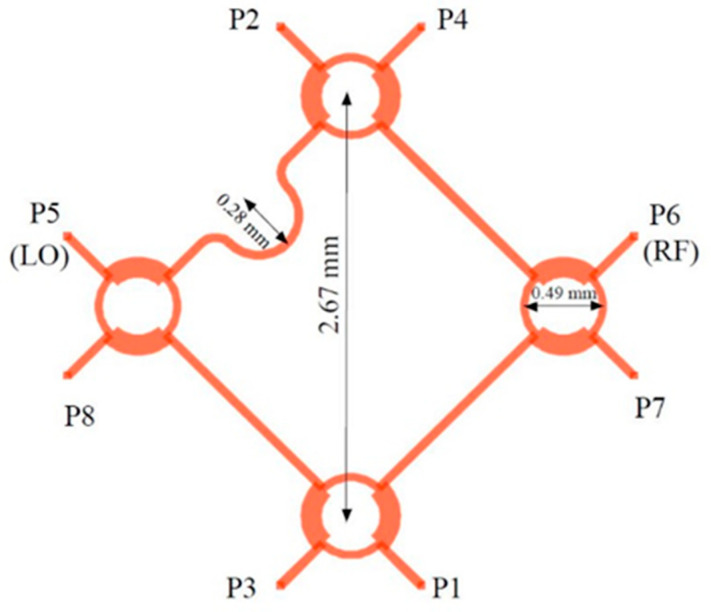
Microstrip multi-port layout.

Details and some dimensions of the multi-port layout are seen in Figure 11. The characteristic impedances of the microstrip lines are 50 Ω (wider line inside the 90° hybrid coupler) and 70.7 Ω for the connection lines. The unused ports P7 and P8 are connected to the integrated MHMIC 70.7 Ω loads, not shown in this layout, and, therefore, the circuit is finally a six-port.

**Figure 12:**
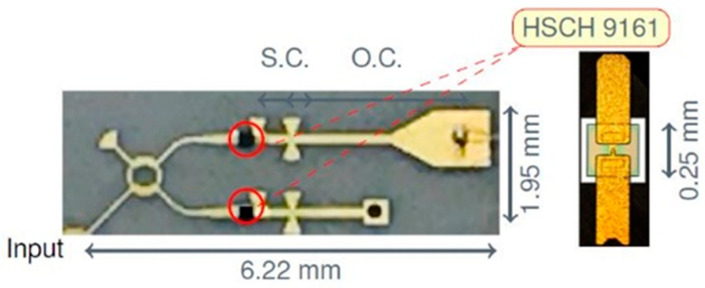
Miniature hybrid microwave integrated circuits (MHMIC) power detector using two HSCH GaAs Schottky diodes.

It is important to mention that, in order to work as an efficient interferometer, power detectors need to be matched almost perfectly over the band. This is directly related to the multi-port theory, as presented in Section 2 of this paper. In a poor matching case, all reflected signals, at any port, will interfere with the incoming signals, and, therefore the output signals will not be accurate anymore. Because of the inherent fabrication errors in the MMIC process, at these very high frequencies, S-parameters of the Schottky diodes are not constant in different fabrication runs. For any integrated interferometer we always choose diodes from the same die, i.e., fabricated at the same time. They will have almost identical S-parameters, however variable from a fabrication run to another. The detector itself is made using a 90° hybrid coupler and two identical diodes. The reflected signals at the Schottky diodes inputs are phase shifted and combined in the hybrid coupler, cancelling each other at the power detector input. The detector will be almost perfectly matched by this procedure, as demonstrated initially for broadband balanced amplifiers design, where pairs of quasi-identical transistors are used in conjunction with hybrid couplers to improve input matching [70]. The detector efficiency will be variable, depending on the MMIC fabrication run, because the diode itself is not matched at all. Open stub reflectors are installed immediately after diodes, cutting the by-passed millimeter wave signal through the parasitic capacitor of the diode. Figure 12 shows details of the power detector layout and of the zero-bias GaAs Schottky diode (model HSCH-9161 of Keysight Technologies [71]).

**Figure 13:**
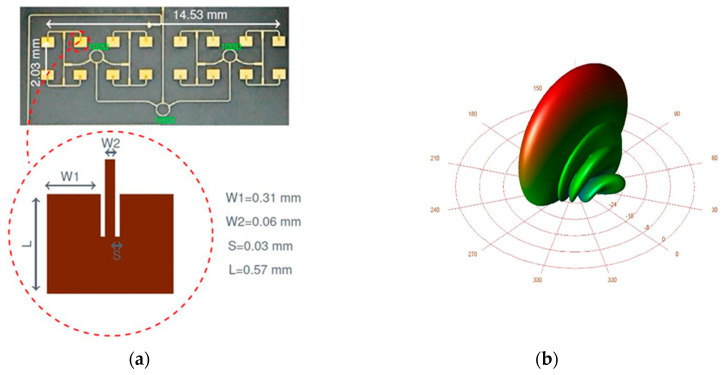
Microstrip antenna array: (**a**) layout; (**b**) simulated 3D radiation pattern.

Layout details of the 16-element antenna arrays are presented in Figure 13. The patch antennas are interconnected by three rounded Wilkinson power couplers and twelve T junctions with related quarter wave matching elements. The gain of each array is around 16 dBi, and the 3 dB beamwidth is around 5° [67].

**Figure 14:**
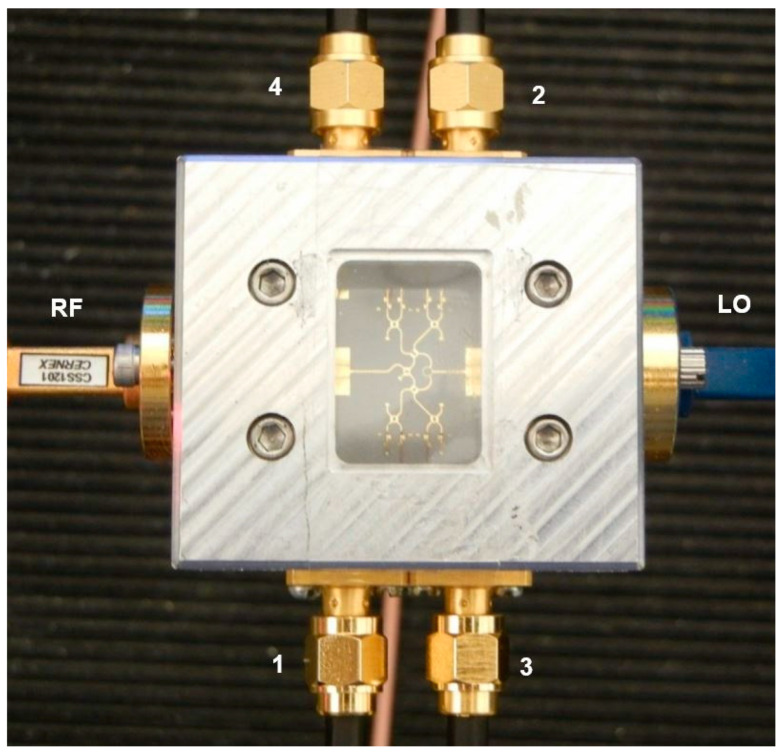
V-band multi-port in aluminum fixture with standard WR-12 flange and SMA connectors.

Figure 14 shows a V-band (57–64 GHz) multi-port, according to the block diagram of Figure 1, with a Wilkinson power divider and three 90° hybrid couplers. It is also embedded in an aluminum fixture with WR-12 flanges for the LO and the RF input and four SMA connectors for the differential quadrature outputs [72]. This circuit can be used in different set-ups for communication and radar sensing over the ISM V-band. Other waveguide components or modules can be connected for system measurements as for example test of LNA, different antenna arrays and so on.

**Figure 15:**
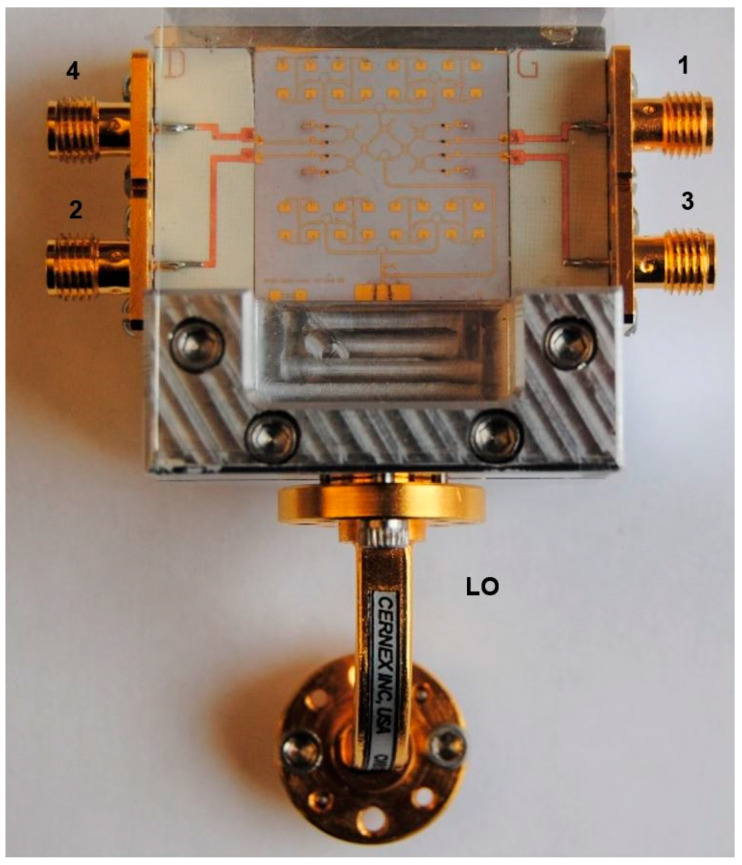
V-band multi-port radar sensor in aluminum fixture with standard WR-12 flange and SMA connectors.

A radar front-end has also been fabricated in the ISM band at 60 GHz using the multi-port circuit of Figure 14. A photo of the radar sensor is presented in the Figure 15. In order to keep the overall size of the circuit smaller than the maximum one allowed by the in-house MHMIC fabrication process, all building blocks and transmission lines have been carefully distributed on the die to avoid parasitic coupling. It is to be noted that on the same ceramic substrate at 60 GHz all circuit dimensions, that are related to the guided wavelength, are bigger with around 30% compared to those at 77 GHz. The directional coupler (bottom, after the RWG/SIW/microstrip transition) has 15 dB coupling. Power detectors use the same architecture as presented previously, in order to ensure a very good matching over the V-band ISM frequencies.

The circuit is intended to be tested in combined radar and communication systems, due to the multi-port interferometer demodulating proprieties, as explained in the test bench measurements of Section 4.

In order to increase the detection range of the radar sensor an integrated LNA must be connected after the receiving antenna array. The LNA die is wire-bonded to the MHMIC front-end in order to reduce the overall size of the radar sensor and inherent transition losses. This interconnection must be carefully done, as indicated by the MMIC manufacturer.

**Figure 16:**
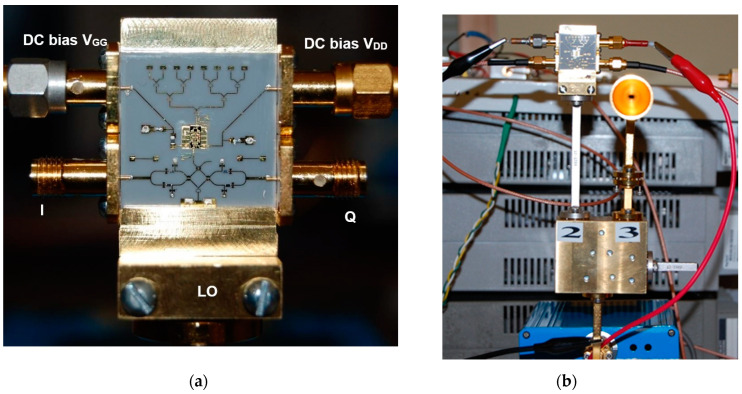
(**a**) E-band multi-port radar sensor receiver in a brass fixture with standard WR-12 flange and SMA connectors; (**b**) complete radar sensor including the waveguide transmitter.

A photo of a 77 GHz radar sensor front-end is shown in Figure 16a [73,74,75,76,77,78]. This sensor architecture does not include the transmitter antenna and combines the multi-port outputs to generate quadrature signals in a quasi-conventional architecture [40]. In this purpose, a 90° hybrid coupler, a delay line acting as a 90° phase shifter, and two diodes connected in opposite directions are used. Output signals are obtained at the lower left/right SMA connectors [78]. The frequency synthesizer is connected via the WR-12 waveguide with a transition to microstrip line input of the multi-port (bottom of the sensor) [79]. The upper left/right SMA connectors are used to bias the LNA. Figure 16b shows the sensor in a complete set-up with a waveguide coupler (connected to its port 2) and a 20 dBi horn antenna to generate the Tx signal.

An integrated 8-element antenna array having a measured gain of 13 dBi is connected to the input of the LNA model HMC-ALH509 of Hittite (now Analog Device) Corporation [80].

The HMC-ALH509 is a three stage GaAs HEMT MMIC LNA which operates between 71 and 86 GHz. It features 14 dB of small signal gain, 5 dB of noise figure and an output power of +7 dBm at 1 dB compression using +2 V V_DD_ supply voltage. The gate voltage V_GG_ is between −0.8 to +0.2 V DC. This LNA is compatible with the conventional die attach methods, as well as thermocompression and thermosonic wire bonding, making it ideal for hybrid microcircuit applications.

**Figure 17:**
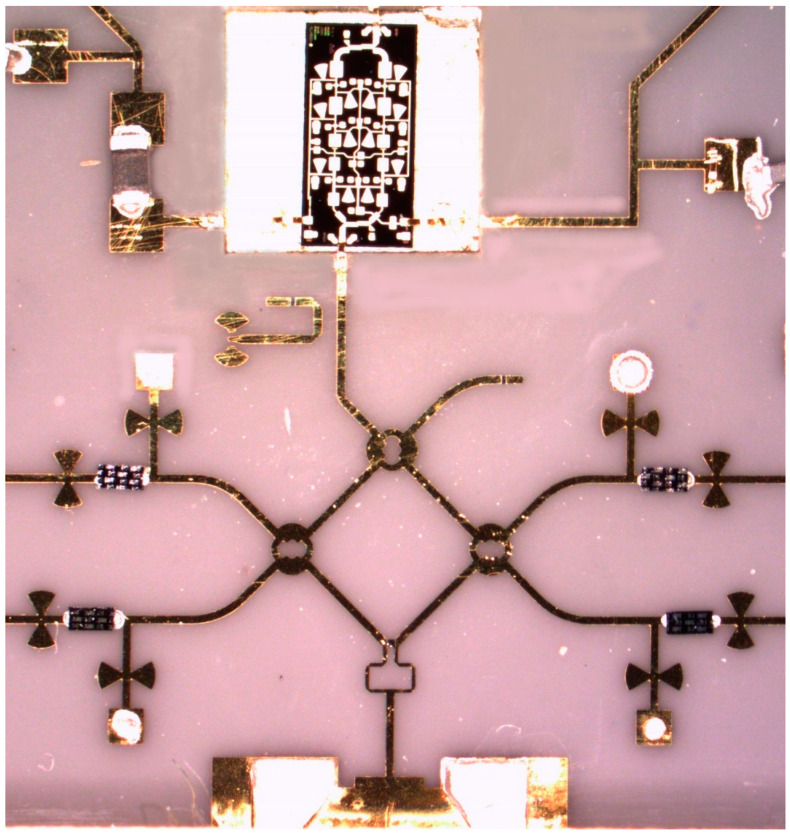
E-band multi-port radar sensor (micro-photograph of bottom part of the MHMIC).

Figure 17 shows a detail of the MHMIC die, including: (i) LNA with input and output wire-bonding and related DC bias network in the top, (ii) the multi-port circuit composed by three 90° hybrid couplers and the Wilkinson power divider (connected to the WR-12 transition in the bottom), (iii) two pairs of Schottky detectors in the left and right side of the multi-port. A rectangular cut into the ceramic substrate is used to insert the MMIC die. The width of the cut is a little larger to make room for the external decoupling capacitors. In this prototype, as recommended by the manufacturer, ribbons of 3 mil length × 0.5 mil width (76.2 µm × 12.7 µm) are used to connect the input to the antenna array and the output to the port 6 of the multi-port. Decoupling capacitors of 100 pF are connected by wire-bonds close to the bias pads to avoid RF oscillations. Both DC inputs are also decoupled with 100 nF capacitors. A 10 Ω series resistor is connected in series to V_GG_ (left of the LNA).

As you can see in the image, a directional coupler is added to allow LNA performance measurements using a probe station with ground-signal-ground (GSG) 150 µm probes. Not shown in this figure, a similar one is connected at its input.

**Figure 18:**
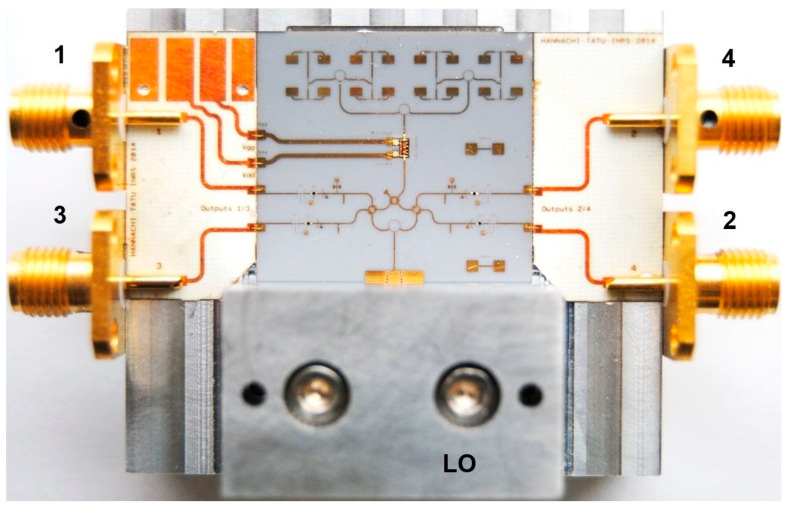
V-band front-end with monolithic microwave integrated circuits (MMIC) low-noise amplifier (LNA) mounted in a rectangular cut in the MHMIC die.

Figure 18 shows another (receiving only) V-band multi (six)-port front-end, using the TGA 4600 MMIC LNA of TriQuint (now Qorvo) Corporation fabricated in 0.15 µm pHEMT technology [81]. The LNA main characteristics are: typical frequency range of 57 to 65 GHz, 4 dB nominal noise figure, 13 dB nominal gain, and bias of 3.0 V, 41 mA. The MMIC chip dimensions are: 1.62 × 0.84 × 0.10 mm (0.064 × 0.033 × 0.004 in). During measurements we have discovered that it would be more practical to connect the LNA in-between two ceramics, not in a cut in the substrate. In the event of a MMIC failure during tests, it would be easier to replace this one.

**Figure 19:**
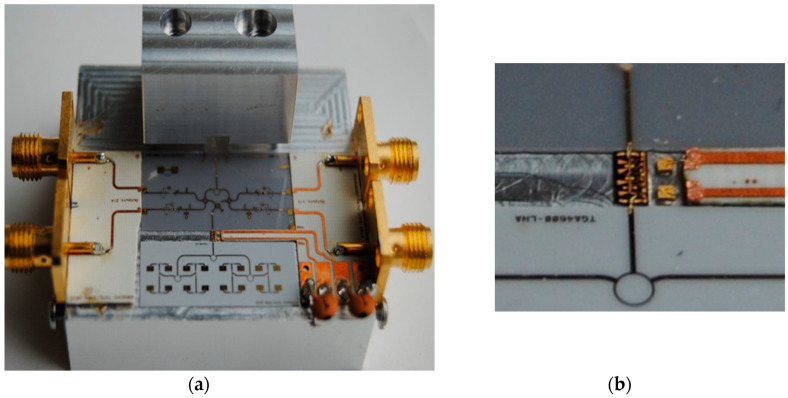
(**a**) V-band front-end with MMIC LNA in-between two separate MHMICs; (**b**) detail of LNA mounting.

Figure 19 shows the same front-end in which the LNA is mounted in-between a ceramic die containing the 16-element antenna array and another one with multi-port and related power detectors connected to the frequency synthesizer via a WR-12 transition to microstrip line. The two DC feed lines of the LNA are printed on a thin PCB section inserted between MHMIC dies. The photo angle allows to better see the top metallic block of aluminum, a half-part of the standard WR-12 to microstrip transition. The bottom part of this transition is embedded in the base of the fixture.

**Figure 20:**
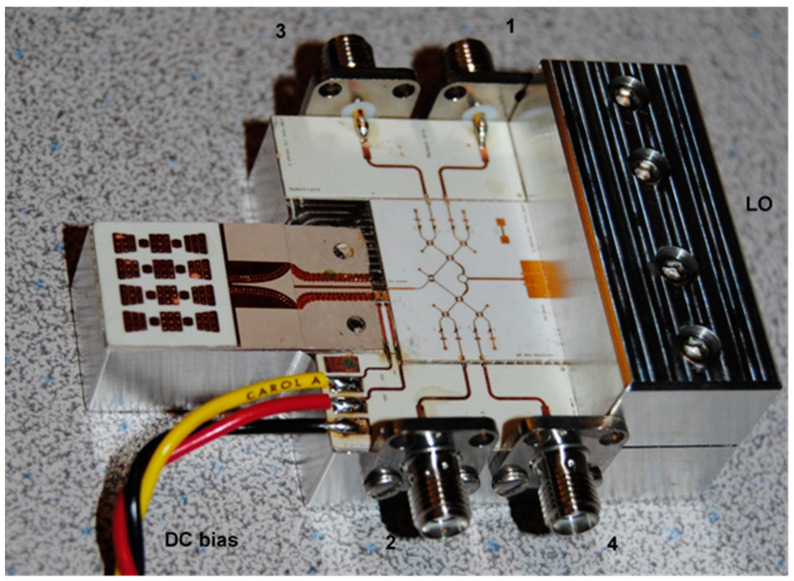
V-band front-end with MMIC LNA in-between two separate MHMICs: overall view.

Figure 20 shows another V-band front-end with a different antenna array and the newest power detector model. The same TGA 4600 is used as LNA. The MHMIC is made on a 254 µm (10 mils) ceramic substrate for more robustness.

**Figure 21:**
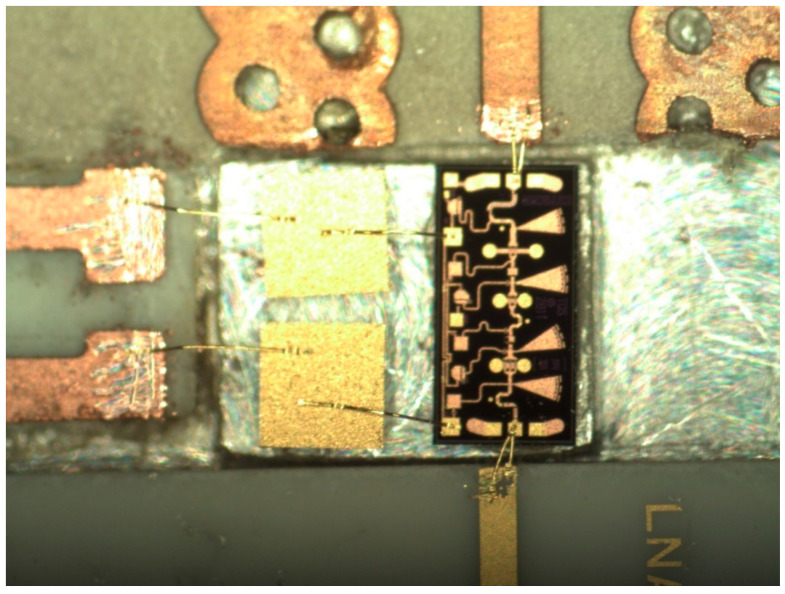
V-band front-end with MMIC LNA in-between two separate MHMICs: detail of mounting.

A detail of the LNA mounting is shown in Figure 21. Two decoupling capacitors are connected directly on the metallic base of the prototype and wire-bonded to the LNA, to avoid unwanted RF oscillations. An eared-aperture coupled patch antenna (EACPA) is connected to the LNA input using a wire-bond [82]. The antenna consists of an array of aperture-coupled-patch (ACP) elements interleaved with an artificial material (i.e., a soft surface structure), which are excited by a strip-line corporate-feed network. This antenna can be alternatively described as an array of 2 × 4 overlapped EACPA. To uniformly excite the elements, an optimized T-junction power divider and some quarter-wavelength transformers are used to construct the feed-network. It has a measured gain of 17 dBi at 61 GHz and more than 15 dBi over the 57–65 GHz band.

A wideband low loss strip-line-to grounded-coplanar-waveguide (SL-to-GCPW) transition is designed and integrated with the designed antenna array. It is employed to match the antenna input impedance to 50 Ω and to facilitate its characterization by connecting to an end-launch mm-wave connector. To minimize the reflection and transmission coefficient responses, the transition at the junction of strip line to GCPW is tapered. Because of the fabrication constraints, two rows of vias are used to shield the transmission lines in this design. The designed transition is well matched and its insertion loss is less than 0.6 dB over the bandwidth. In addition, it does not degrade the radiation pattern of the proposed antenna array when it is integrated with it.

After more than 15 years of expertise in the embedded technologies in millimeter wave prototyping, we can conclude that, for initial lab validation using standard equipment with RWG flanges, the use of the all metallic waveguide structures is costly and the prototypes are extremely heavy. We therefore recommend the use of a mixed technology in which a minimum number of transitions are used to connect to the standard laboratory equipment, such as frequency synthesizers or millimeter wave heads of the Vector network Analyzer (VNA). In order to reduce the number of transitions, integration of antenna arrays is preferred. A very good RWG transition includes usually a SIW section, the optimal interface between a standard waveguide and a planar integrated one. MHMIC technology is used for all other passive elements on the one (no LNA) or two dies (with embedded state-of-the-art LNA MMIC connected in between MHMIC dies of the antenna array and down-converter). If a broadband response is requested, the use of a perfect symmetrical structure of the quadrature down-converter, with a broadband Wilkinson power divider is preferred. This is possible only in MHMIC technology where resistors can be integrated. Broadband matched power detectors using identical pair of diodes and a 90° hybrid coupler are the preferred power detectors, ensuring a quasi-perfect match over the frequency band.

## 4. Circuit and System Characterization Techniques

A very important step in front-end design is circuit and system characterization. In the last section different fabricated front-ends have been presented. Before fabrication, after the circuit design using the appropriate software, it is crucial to measure and characterize all MHMIC and RWG circuits, to choose the best ones for the prototype fabrication. A second step will be to characterize the prototype in an appropriate system test-bench using the available equipment of the laboratory.

### 4.1. Circuit Characterization

#### 4.1.1. S-parameter Measurements

For the S-parameter measurements with a two-port VNA, the symmetry must be fully exploited to reduce the number of fabricated two-port circuits on a single ceramic die (size 2.54 × 2.54 cm).

**Figure 22:**
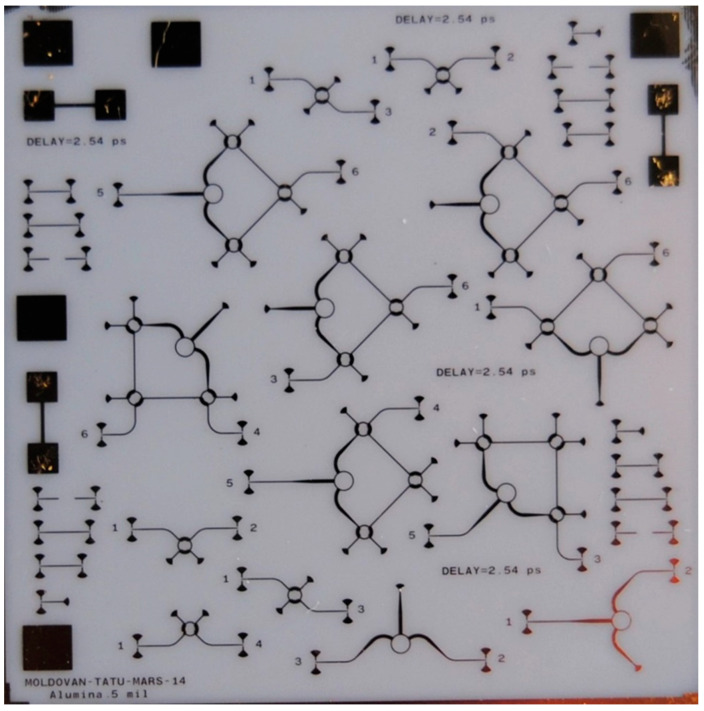
Photo of MHMIC circuits on the ceramic die prepared for full-port S-parameter measurements.

A photo of a complete MHMIC die for circuit characterization is presented in Figure 22. This is an example of a W-band circuit characterization on a 127 µm (5 mils) ceramics. For example, full S-parameter measurements of symmetrical three-ports (such as Wilkinson power divider) require two two-port printed circuits. For four-ports (such as hybrid couplers), three different circuits are needed. Finally, for the proposed multi-port, due to its symmetry, measurements require seven different two-port printed circuits (instead of fifteen, for general combination of six ports to be measured into sets of two). All unused ports are loaded using integrated resistors of characteristic impedance value. Via-holes are avoided for grounding in order to reduce parasitic elements and to ensure the measurements’ repeatability [40,83]. Broadband RF short circuits implemented with quarter waveguide open stubs are used instead. As usual, transitions to coplanar lines are required to perform VNA measurements using the probe station.

The thru-reflect-line (TRL) calibration kit is implemented on the same die. This is mandatory to obtain a successful and repeatable calibration. Calibration using coplanar probes is very challenging at these high frequencies on the MHMIC prototype, as detailed further. Several TRL kits are fabricated on the die, to be used in case of damages occurred when landing the probes, as seen in Figure 22. The characteristic impedance was chosen at 70.7 Ω to keep the optimal aspect ratio of a short microstrip transmission line. The calibration kit line (L) length must be shorter than half of the guided wavelength at the maximal frequency, to avoid phase ambiguities during calibration. The delay corresponding to this line is used in the VNA calibration kit description, required before starting the calibration procedure.

This works demonstrated that, despite the claims of some experts, microstrip technology can be successfully used at these higher frequencies by appropriate choice of a good quality, very thin substrate, transmission line characteristic impedances, and components’ shape. Authors claimed that, when fabricated in 2014, this was the first MHMIC multi-port operating at more than 80 GHz [83].

**Figure 23:**
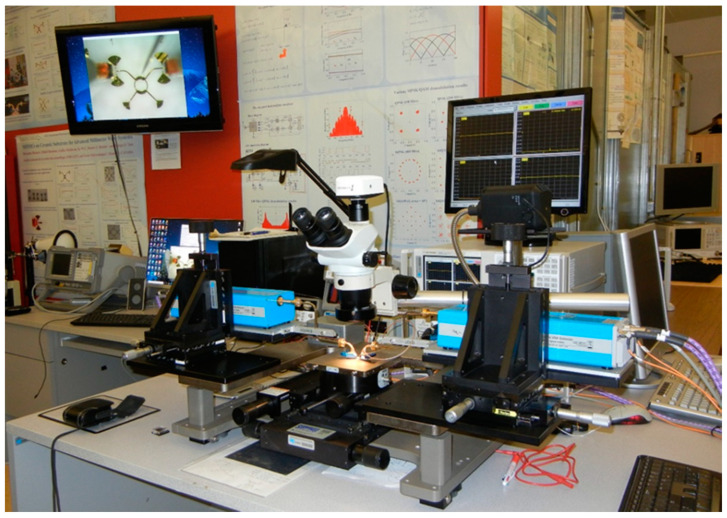
Measurement set-up: millimeter wave probe station and vector network analyzer (VNA).

Figure 23 shows the photo of the measurement set-up. It was built in-house using equipment and parts from different manufacturers to best comply with our technical requirements. The set-up is mainly composed of a millimeter wave VNA and a high-precision manual probe station.

The VNA includes the E 8362 PNA Network Analyzer and the N 5260A Millimeter Wave Head Controller from Keysight (Agilent, Santa Clara, CA, USA) Technologies. Two-millimeter wave extenders, from OML Inc., Campbell, CA, USA, optimized for Keysight (Agilent) N 5260A, are used for WR-12 waveguide operation. Considering the huge attenuation on millimeter wave cables, this ensures very high dynamic range measurements. The measurement system uses a modified Cascade Microtech Analytical probe station, Summit 9000. Two bended WR-12 waveguides are connected to millimeter wave extenders. Cascade Microtech GSG wave guide probes having a pitch of 150 µm are used. The precision manual positioners are fabricated by Focus Microwaves Inc., Montreal, QC, Canada. The positioners allow easy and accurate movements on three axes. The highest precision is on Z axis for probe landing, where sub-0.1 µm resolution is required. The set-up includes an Olympus SZ61 stereo microscope equipped with a high-resolution Infinity 1 camera. The Infinity capture and the analyze software allow precise measurement of the circuits’ physical dimensions, with around 1 µm resolution. Images are also displayed on a 32-inch LCD monitor making probe positioning more convenient.

**Figure 24:**
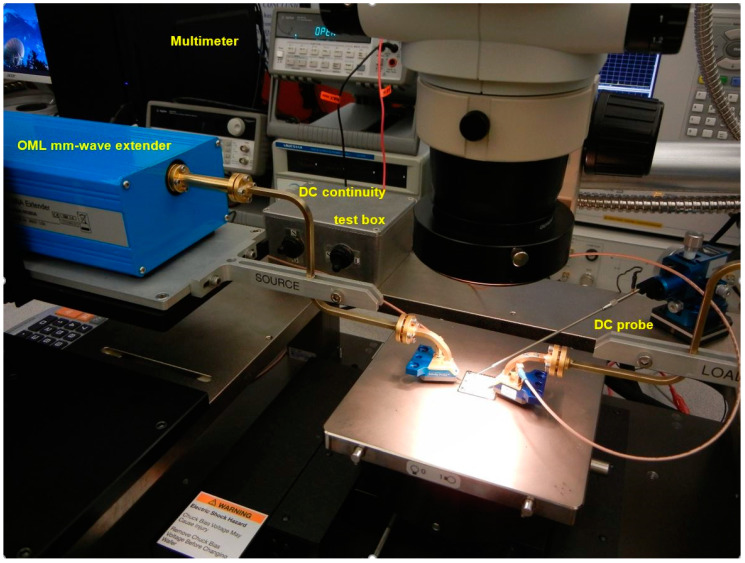
Measurement set-up: MHMIC circuit under test, DC continuity test box, probes.

**Figure 25:**
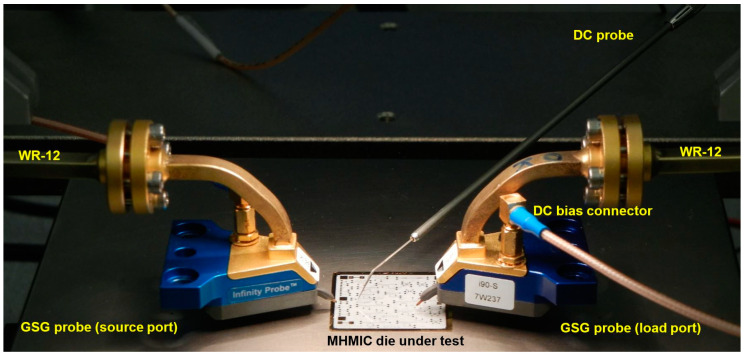
Details of measurement set-up: MHMIC circuit under test, GSG and DC probes.

Details of the S-parameter measurements set-up, including a MHMIC die under test, are presented in Figure 24 and Figure 25.

Repeatable and precise S-parameter measurements (magnitude and phase) require that the same pressure be kept on coplanar probes and that the probes be landed at the same place at the coplanar inputs, from a two-port MHMIC to another. Multi-port characterization requires up to 15 two-port measurements. The maximum number of 15 represents a combination of 6 samples (ports) by 2 objects (VNA inputs). Depending on the symmetry of the circuit, this number can be reduced, for example to 7, for the multi-port in Figure 22. The S-parameter measurement is very challenging, with circuit dimensions at the limits of the fabrication and measurement process.

**Figure 26:**
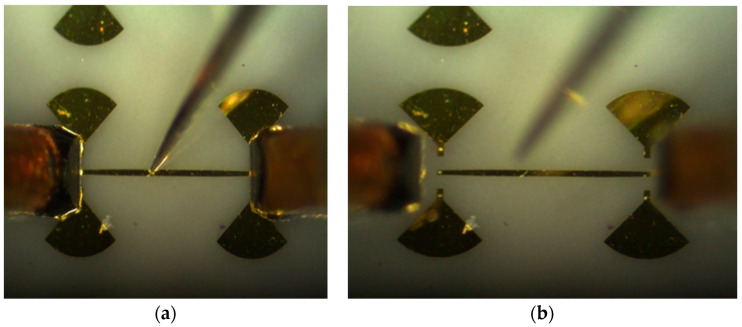
Coplanar and DC probe landings on calibration kit line: (**a**) down; (**b**) up after measurement.

Figure 26 photos show details of the coplanar GSG probe positioning, including traces of contact points (footprints) after measurements. As mentioned before, the MHMIC conductive gold layer is 1 µm thick; the probe must land on it without scratching away the gold. A minimal footprint after landing will allow a reuse of the calibration kit. Therefore, probe landing requires an extreme precision. For a successful TRL calibration all consecutive six landings (two on THRU connection, two on opens for REFLECT, and two on LINE connection) must be almost perfect.

In order to keep the contact pressure at a constant and optimal value, additional Cascade Microtech DC probes and a precision digital multimeter are used to measure the contact resistance between coplanar probes and the transmission lines, as seen in Figure 24, Figure 25 and Figure 26. The procedure, developed in-house, requires the use of coplanar probes with a DC bias connection. For each probe we have therefore access to each separate ground (G) or signal (S) connection on source (S) or load (L) millimeter wave module port of the VNA. Usually a value of up to 3 Ω is targeted for each landing. At the first contact of the GSG probe, the measured resistance is in the range of hundreds of Ω. A movement on the vertical direction fractions of µm closer will ensure a good contact of the probe. As noted, the conductive gold layer is only 1 µm thick, and, if damaged, the contact resistance increases again, making measurement difficult or impossible on the same coplanar port. Of course, the DC probe is lifted during the S-parameter measurements.

**Figure 27:**
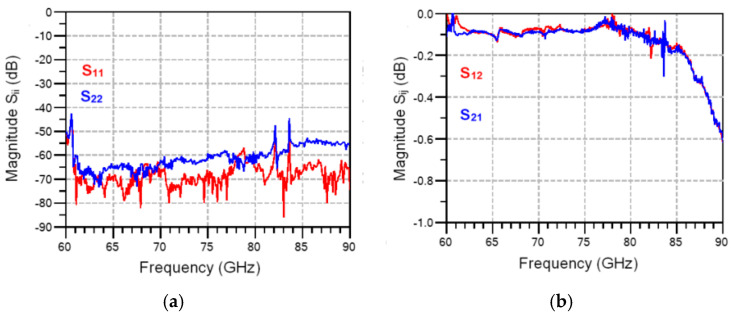
Initial calibration results on line (L): (**a**) matching; (**b**) insertion loss.

**Figure 28:**
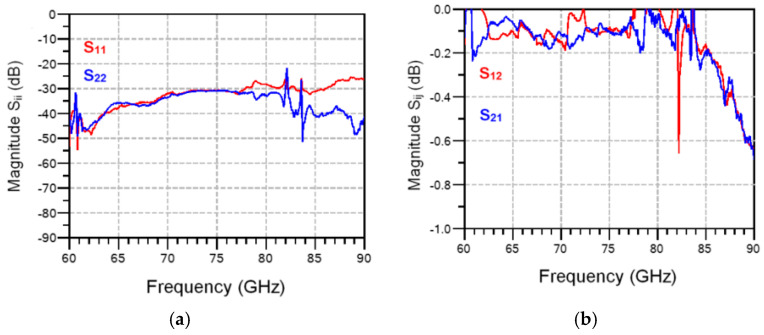
Calibration kit line (L) measurements, second landing after 5 h of measurements: (**a**) matching; (**b**) insertion loss.

The time required to perform a very good calibration (60–90 GHz, IF bandwidth of 10 Hz, 601 points) is usually more than 60 min, after a warming up of the entire system of at least 30 min. Values of 50 dB and fractions of dB are targeted for return and insertion losses on the line of the calibration kit respectively, as seen in Figure 27. At the end of a measurement day, 5 h after the initial calibration, the time required to measure all circuits on a MHMIC die, we repeated the measurements on the same transmission line. Slight degradation of results, as seen in Figure 28, is due to the warming of the system and a second landing on the same transmission line. However, insertion losses are almost the same, while return losses are still acceptable.

Precision probes positioning is also particularly important for measuring the transmission phase. For example, a shift of 4.25 µm along the transmission line corresponds to 1° phase error in measurements on a 5 mils ceramic substrate, 70.7 Ω characteristic impedance line, at 77 GHz. Please note that probe pitch is 150 µm, and 4.25 µm corresponds to 3% of this pitch. This is particularly challenging for a multi-port measurement where the phase is a crucial parameter. Measurements of the multi-port in Figure 22 require 14 probe landings with extreme precision.

**Figure 29:**
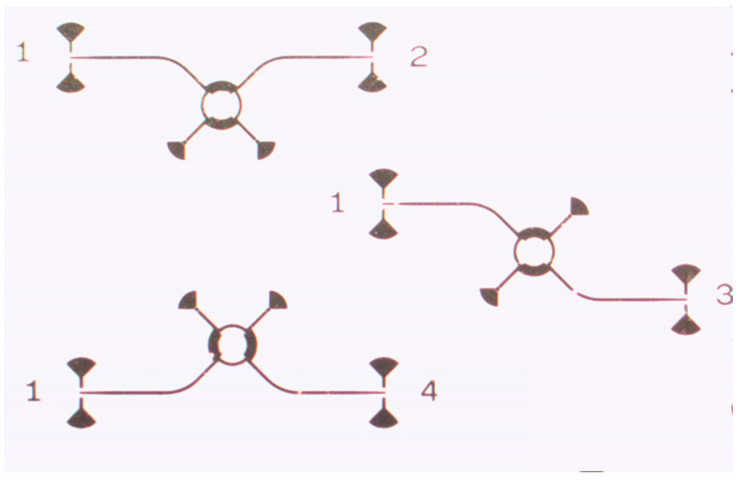
MHMIC 90° hybrid coupler prepared for measurements.

The measurement layouts of a 90° hybrid coupler, part of the MHMIC die of Figure 22, are shown in the micro-photograph of Figure 29. The circuit operates over a 10 GHz band having its center frequency at 86 GHz. Because of the coupler symmetry, only three different layouts are needed instead of six, as for a general four-port. As usual, all unused ports are terminated by matched loads connected to a RF short-circuit made with an open stub.

**Figure 30:**
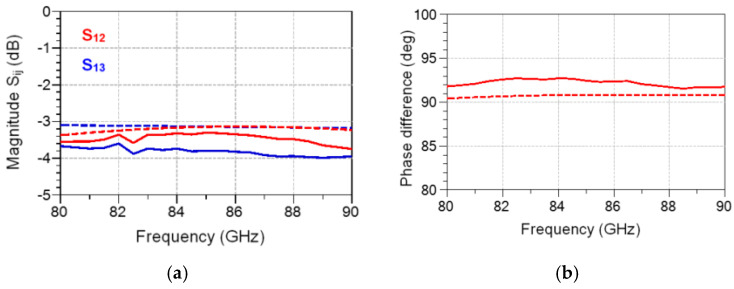
MHMIC 90° hybrid coupler: (**a**) transmission; (**b**) phase difference.

**Figure 31:**
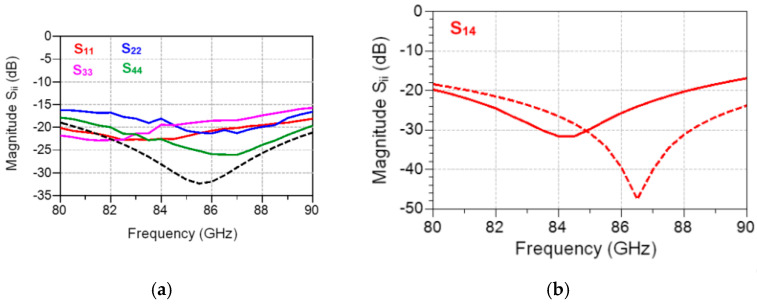
MHMIC 90° hybrid coupler: (**a**) matching; (**b**) isolation.

Simulations (dotted lines) and measurements (continuous lines) of a 90° hybrid coupler part of the MHMIC die of Figure 22 are presented in Figure 30 and Figure 31. Excellent values of power split, 90° phase difference, return loss, and matching are obtained in the design process, as shown by the dotted lines. All return losses at the four ports are equal and superposed, as seen in Figure 31a.

Measurements, displayed with continuous lines in the same figures, are in good agreement with the simulations. Some measurement errors can be however observed. Firstly, the glitches at 82 and 84 GHz, specific to our in-house measurement system, are eliminated by initial calibration, but they appear after a warming up of several hours, as seen in Figure 28. Secondly, some small errors appear in the phase measurements. As indicated previously, a 4.25 µm positioning error of the GSG probe along the transmission line corresponds to a 1° measurement phase error. Therefore, phase error measurements of maximum 2.5°, as shown in Figure 30b, are explained by around 10 µm positioning error of the 150 µm GSG probes in the four consecutive probe landings required for this measurement, a very good measurement performance with a manual probe station.

**Figure 32:**
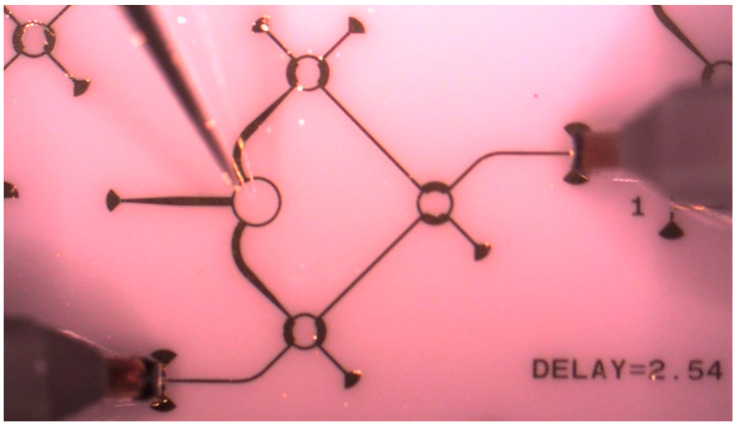
Multi-port under measurements (S_36_).

Figure 32 shows a multi-port under measurement, in which two GSG probes and the DC probe are used, as explained previously. This is part of the total of seven two-port measurements required to build its computer model.

To conclude S-parameter measurement description we notice that precise measurements are mandatory for building accurate computer models used in advanced system simulations, but as seen, they are very challenging in practice.

#### 4.1.2. Measurement Based Computer Models and Circuit Characterization

In order to perform system simulations with realistic behavior, computer models based on S-parameter measurements must be implemented into design and analysis software, for example the advanced design system (ADS) of Keysight Technologies. All two-port VNA S-parameter measurements of the MHMIC multi-port are imported as Data Access Components (DAC) [83].

**Figure 33:**
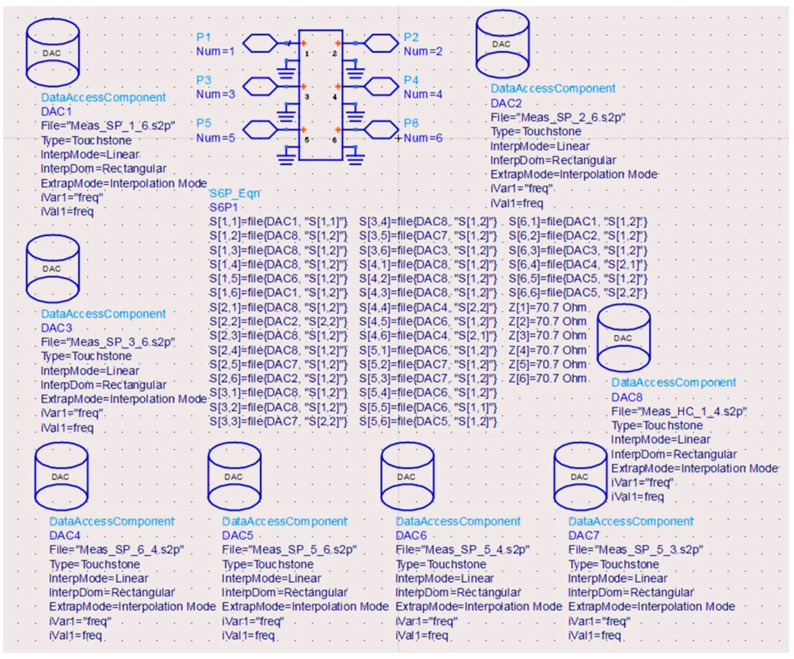
Advanced design system (ADS) multi (six)-port circuit model based on S-parameter measurements.

Figure 33 shows the multi-port model as generated into ADS, based on two-port S-parameter measurements on the MHMIC die, that are stored on different s2p files. The name of the files contains the circuit port number connected at the source of the VNA (1) and at the load port of the VNA (2) to avoid any confusions. An accurate model requires an extreme measurement precision, on multiple GSG probe landings, as detailed in the previous section.

A second method to generate a multi-port model is to use its building blocks: Wilkinson power divider, 90° hybrid couplers, and transmission lines. This allows testing of different multi-port architectures in simulations and obtaining models with S-parameter measurements much more easily.

It is to be noted that it is generally difficult to evaluate a multi-port interferometer performance using only S-parameters. Its four output signals are a vectorial combination of two inputs: the reference signal at the LO port and the input unknown signal at the other port. Therefore, it is not sufficient to evaluate matching, transmission losses, and phases separately. It is suggested to use several global performance analyses: (i) harmonic balance and the *q*_i_ points for general circuit evaluation, or (ii) homodyne quadrature demodulation and EVM for radar and communication systems.

**Figure 34:**
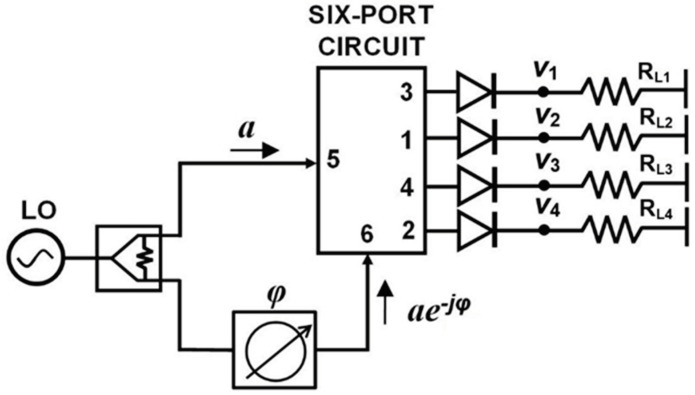
Harmonic balance simulation simplified block diagram.

A harmonic balance analysis is performed as an initial design validation of interferometric capabilities of the multi-port circuit. The block diagram of harmonic balance simulation is presented in Figure 34. The millimeter wave signal generated by a local oscillator LO is split into two equal parts: the reference signal, injected at port 5, and the phase shifted one, injected at port 6. Ideal power detectors are connected to circuit outputs in order to highlight only multi-port performances. In practice, matched Schottky detectors are used, as presented in Section 3 of this paper. Output detected voltages Vi are generated on load resistors R_Li_. High-impedance loads are recommended to increase output voltage values, because diodes are current generators.

**Figure 35:**
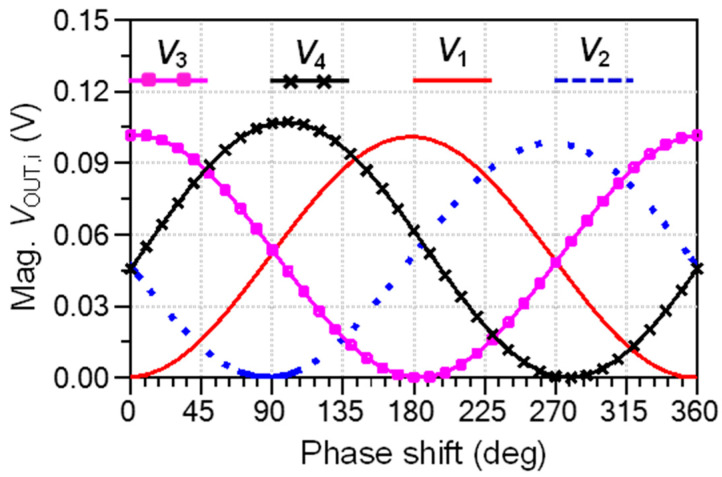
Magnitude of the power detected output voltages (85 GHz).

Ideally, if the phase φ is swept over 360°, each output detected voltage varies as a shifted sinus function, as described in the six-port theory [38,40] and resumed in Equations (6) and (7). The LO power is set at 0 dBm for convenience and the phase is swept from 0° to 360°. The displayed waveforms in Figure 35, having quasi-sinusoidal shapes are in very good agreement with the theoretical equations. Minimum values are close to zero and are shifted by around 90° multiples. Maximum values of all signals are almost the same. As observed V_1_ and V_3_, and, respectively, V_2_ and V_4_, are in opposition of phase, and voltages with odd and even indexes are shifted with 90°. Therefore, differential quadrature output signals can be obtained in multi-port down-conversion with this circuit.

From a historical point of view, the first six-port circuits were used as alternative, low-cost, vector analyzers [1,2,3,4,5,6,7]. In those designs, the power of one of the outputs is always proportional to the reference signal. The other three output detected powers are function of the reflection coefficient of the device under test (DUT). By definition, the q_i_ points are the values of the measured reflection coefficient which nulls the detected power at port i. For these circuits, the q_i_ points are ideally spaced by 120° in the complex plane. In order to obtain the DUT reflection coefficient (Γ_L_), three circles having their center in the q_i_ points and radiuses related to the measured detected power are plotted. The Γ_L_ is the intersection point of these circles [2].

By analogy, the q_i_ points for multi (six)-ports used in receiver front-ends are defined as the normalized complex value of the input RF signal at port 6 which minimizes the measured power at the i = 1 to 4 outputs. If the input RF powers at ports 5 and 6 are equal, this minimal value is theoretically equal to zero. Fast analog signal processing of millimeter wave signals, without any calibration, requires four q_i_ points spaced by 90°, because there is no reference output power [40]. The output power levels function of phase shift can be obtained using the block diagram of Figure 34:

P_i_ = {mag [aS_5i_ + aexp(−jφ) S_6i_]}^2^ = [mag (a)]^2^ [mag (S_5i_ + q_i_ S_6i_)]^2^ (8)

q_i_ = −S_5i_/S_6i_, i = 1,2,3,4 (9)

**Figure 36:**
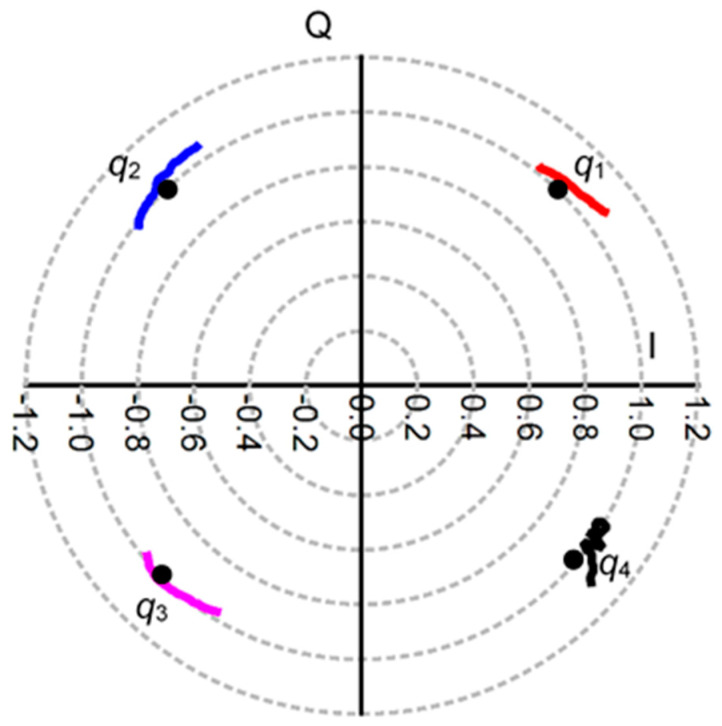
The *q*_i_ points position of the multi-port over 10 GHz band (80–90 GHz).

The four q_i_ point positions over the 80–90 GHz band, obtained using S-parameter measurements of the multi-port circuit in Figure 22, are presented in Figure 36.

The ideal position of each point, on the unit circle, is marked by a dot. As seen, measurements of the circuit confirm that the q_i_ points are clearly separated by quasi 90° multiples and are very close to the ideal values. A rotation movement of around 20° is observed from 80 GHz to 90 GHz. If frequency is increased, the q_i_ points will rotate in clockwise direction. This is mainly related to the variation of electric length on transmission lines versus frequency. It is to be noted that rotations are usually compensated in multi (six)-port receivers, just like all other phase shifts, for example due to cable lengths or to propagation or Doppler effects, by using a phase shifter at LO port or by signal processing [40].

#### 4.1.3. Antenna Measurements

Previous circuit analysis methods are therefore used to ensure that the multi (six)-port circuits are working properly, and that they can be used in front-ends. It is obvious that all other components must be tested before integrating them in the prototypes. It is particularly the case of antenna arrays that are tested in anechoic chambers. In order to prepare the antenna for measurement, a specific connector must be added for millimeter wave measurements in the 60–90 GHz band: waveguide (WR-10 or WR-12) or coaxial (1.85 or 1 mm), depending on the central frequency and the required bandwidth.

**Figure 37:**
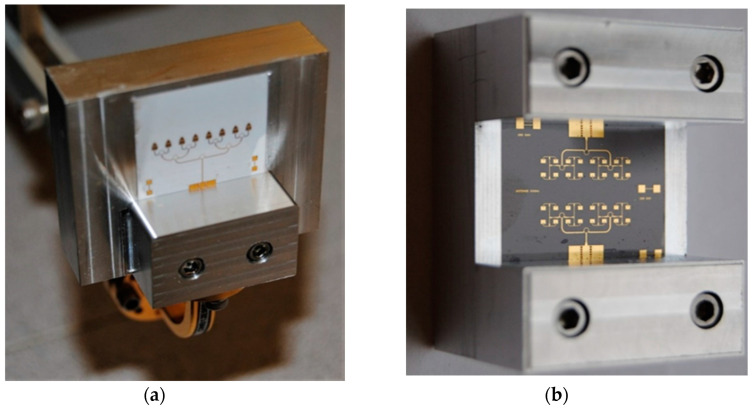
Antenna arrays with WR-12 connections for testing: (**a**) 8-element V-band; (**b**) 16-element W-band.

Figure 37 shows two MHMIC antenna arrays on a 127 µm ceramic substrate prepared for measurements using WR-12 standard connectors. These metallic fixtures can be used in anechoic chambers, in VNA waveguided measurements or in test benches where a separate antenna connected in standard waveguide is required. Figure 37a shows a V-band 8-element antenna array using patches and a gap-coupled parasitic triangular element (used for beam tilting from perpendicular to the die toward horizontal [84,85]). A gain of 12 dBi is measured at 61 GHz, and for the band 60–63 GHz this is more than 10 dBi. Simulations show only around 1.5 dB more gain over the band. Both measured and simulated return losses are better than 15 dB over the same 3 GHz band. Figure 37b shows two W-band 16-element antenna arrays. Both radiation pattern and parasitic coupling between arrays can be measured with this particular lay-out. The parasitic coupling between arrays is an important parameter in bi-static radars where different antennas are used for transmission and reception on the same die, as seen in various prototypes described in Section 3 of this paper.

**Figure 38:**
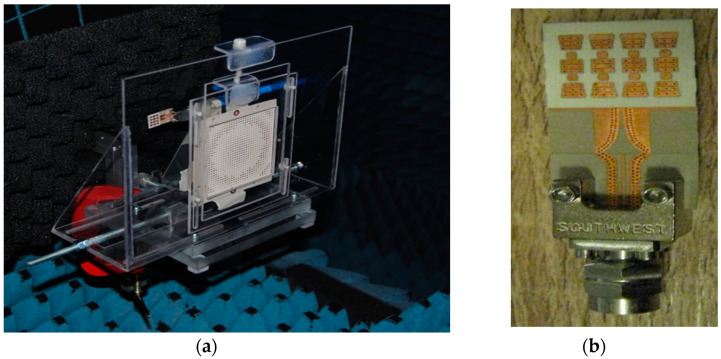
V-band antenna array measurement: (**a**) mounting in anechoic chamber; (**b**) feed antenna.

**Figure 39:**
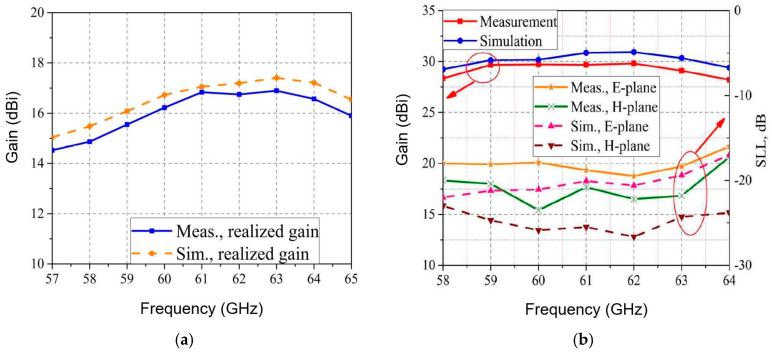
V-band antenna array measurement: (**a**) gain of the feed antenna; (**b**) gain of antenna array.

Figure 38a shows a detail of the V-band high gain planar lens antenna array in anechoic chamber. The feed antenna, that is part of the front-end of Figure 20, is connected using a 1.85 mm reusable end-launch connector, as seen in Figure 38b. This antenna system utilizes the spatial feeding technique and the physical optic principles to obtain efficient collimated or shaped beam [86,87]. Due to the planar lens antenna array, the measured gain of the antenna system passes from around 17 dBi for the feeder itself [82] to around 30 dBi at 61 GHz [87], as seen in Figure 39a,b. This gain is more than 27 dBi from 58 to 64 GHz. In both E and H planes the measured 3 dB beam is around 3°, while the 10 dB beam is around 7°. Secondary lobe levels (SLL) are around 20 dB less than the main beam at 61 GHz [60]. Therefore, this high gain antenna system is a viable alternative for the antenna required in point to point communications over the millimeter wave V-band.

### 4.2. System Characterization

Various test-benches have been implemented for multi-port and radar system testing. Because all described prototypes have a standard RWG input, the connection with millimeter wave sources is straightforward. In addition to S-parameter measurements, a multi-port can be tested in different system test-benches, as you can see in the next examples. If both millimeter wave inputs have standard waveguide connections, the set-up will allow the characterization of the circuit itself (a broadband waveguide antenna or direct waveguide connection can feed the input ports).

#### 4.2.1. Multi-Port as Direct Quadrature Demodulators

- Test-Bench #1

**Figure 40:**
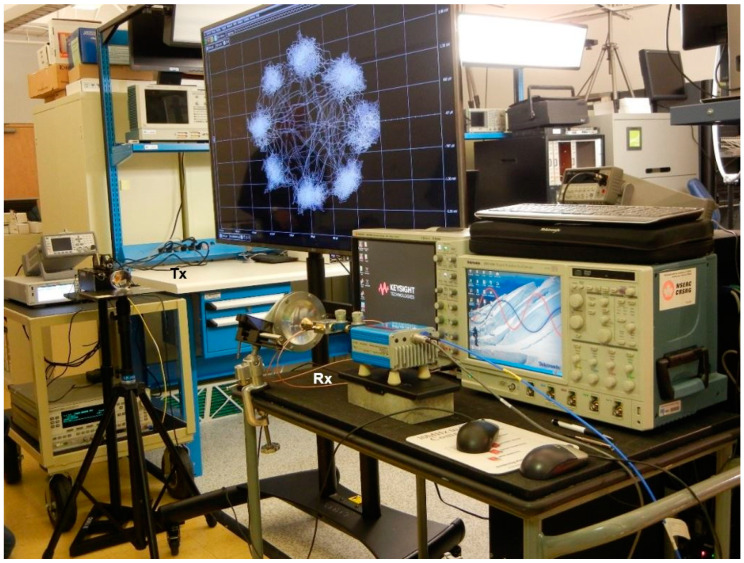
V-band multi-port characterization test-bench using modulated signals.

Figure 40 shows a test bench using the multi-port as a direct quadrature demodulator. A V-band transmitter is used to generate the PSK/QAM signals to be demodulated at the receiver side using the multi-port circuit. The transmitter uses a Virginia Diodes Signal Generator Extension Module (SGX) -VDI Model: WR12SGX [88] fed by a microwave unmodulated signal of 10 dBm and an IF modulated signal of 0 dBm from a Keysight MXG Vector Signal Generator model N5182B. For example, to generate a 61.2 GHz modulated signal, a 10 GHz unmodulated signal (that will be multiplied by 6) and a 1.2 GHz modulated signal are the inputs of the VDI module. The transmitter power is around 10 dBm. A 20 dB waveguide antenna and a dielectric lens are also used to increase the gain. It is to be remembered that according to the Friis equation for the free space attenuation A_FS_ [89], a 68 dB loss is expected at 1 m distance between antennas at 60 GHz.

A_FS_ = 20log(λ/4πd) (10)

In the previous equation λ is the free space wavelength, and d is the distance between antennas.

In order to evaluate the power at the input of the receiver, we can use the Friis equation for received power P_Rx_ (dB), where P_Tx_ is the power delivered to the terminals of an isotropic transmitting antenna (dB), G_Tx_ is the isotropic gain of the transmitting antenna in the direction of the receiving antenna (dBi), and G_Rx_ is the isotropic gain of the receiving antenna in the direction of the transmitting antenna [89]:

P_Rx_ = P_Tx_ + G_Tx_ + G_Rx_ + A_FS_ (11)

**Figure 41:**
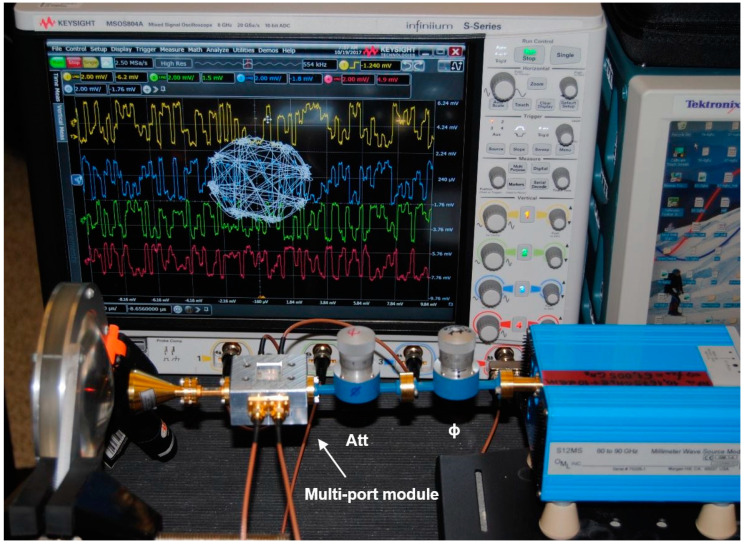
V-band multi-port characterization using modulated signals: detail of the down-conversion.

The V-band multi-port module of Figure 14 is under test in reception. At the RF input (port 6) the same cylindrical horn antenna of 20 dBi gain, as for the transmitter, and another dielectric lens is used. The combined gain of lenses is around 15 dB; they are used because this set-up does not include any amplifier (LNA or baseband). The multi-port is fed at the LO port by an OML S12MS millimeter wave source module (x6) having an output V-band signal of around 7 dBm [90]. Its input signal frequency is 61.2 GHz/6 = 10.2 GHz at 10 dBm. A 40 dB variable attenuator is used to reduce the LO signal at levels comparable with the port 6 input, where in this case it is estimated at −3 dBm for a perfect antenna and lenses alignment. The in-series phase shifter allows the rotation of the constellation on the screen, giving additional hardware flexibility to the set-up. For displaying the demodulated constellations and the quadrature differential signals, a Keysight Infiniium S-series MSOS804A mixed signal oscilloscope connected to a TV screen is used, as seen in Figure 40 and Figure 41. The 1 MΩ impedance inputs are required to offer a high impedance load at the integrated power detectors outputs, to increase the detector output voltage levels.

Various PSK/QAM signals have been demodulated with the set-up. Captured on the screens of Figure 40 and Figure 41, it can be observed that all eight symbols of the 8PSK constellation are arranged quasi-equidistant on a quasi-perfect circle. In addition, the two pairs of quadrature output signals (1,3 and 2,4) from top to bottom in Figure 41) are phase opposite (differential outputs) and with comparable magnitudes. All results are in accordance with the theoretical Equations (1)–(7) of Section 2, validating the multi-port performances in a different way than by S-parameter measurements or q_i_ points analysis. It is to be noted that power detectors are included in the prototype, therefore, the demodulation results on test bench prove also their almost identical performances and very good matching. LNA and band-base amplifiers will be connected to the multi-port module to build a more performant receiver, as it can be seen in the next set-up.

- Test-Bench #2

**Figure 42:**
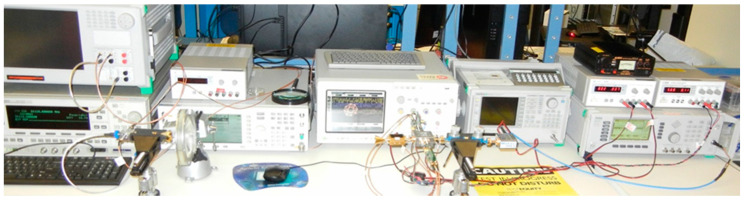
V-band multi-port test bench (version II).

Figure 42 shows a second version of the V-band test-bench, using the same multi-port circuit and different other waveguide modules (commercial LNA, home-made frequency multiplier, V-band I/Q modulator) and four high-speed baseband amplifiers.

This second set-up was built to verify the capabilities of a V-band front-end with MHMIC circuits embedded in separate modules. This allows testing of such modules, in order to choose the best available MMIC for further integration of a front-end, in both transmitter or receiver sides.

**Figure 43:**
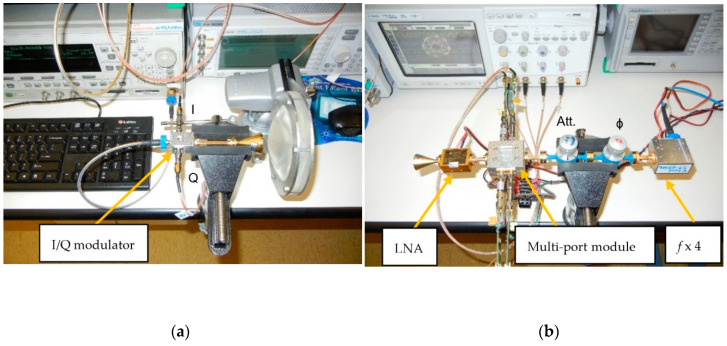
V-band multi-port test-bench: (**a**) transmitter side; (**b**) multi-port receiver side.

In the transmitter side, a MMIC I/Q direct modulator is used. As seen in Figure 43a, the Analog Devices MMIC type HMC MDB 218 GaAs sub-harmonic mixer (54–64 GHz) [91] is embedded in a home-made fixture, as described previously for the multi-port. It is fed by a 10 dBm millimeter wave signal having half of the V-band frequency signal. The baseband I and Q signals are generated by a Keysight E4438C Vector Signal Generator. The V-band modulated signal power is around 0 dBm. A cylindrical horn antenna of 20 dBi gain is connected to its output and a dielectric lens is used to increase the transmitter gain with around 10 dB.

In the receiver side, as seen in Figure 43b, more modules are connected to the multi-port. A commercial V-band low noise LNA fabricated by SAGE Millimeter Inc., having 20 dB gain and noise figure of 8 dB, is connected between the receiving horn antenna and the multi-port module input [92]. The LO signal is generated with a home-made frequency multiplier circuit (4×). This multiplication coefficient is obtained in two stages: (i) the input frequency is doubled using an GaAs MMIC 2× active frequency multiplier, the HMC578 (24–33 GHz output) [93], and (ii) the frequency is doubled once again with an GaAs MMIC 2× passive frequency multiplier, the HMC 1105 (20–40 GHz input) [94]. From a V-band frequency/4 of around 3 dBm power, an equivalent in power V-band signal is obtained at the module output. In the current set-up, a CW 15 GHz signal is multiplied by 4 to obtain a 60 GHz CW one. Waveguide attenuators and phase shifter are used at LO port as usual.

Home-made baseband amplifiers (BBA) built with ultra-high-speed operational amplifiers (AD8000, f_T_ = 1.5 GHz) are connected to multi-port module outputs. The main characteristics are: 20 dB gain, impedance input of 4 KΩ (a load value compromise; a high impedance required by integrated power detectors and a low impedance required by higher data-rates), and the output impedance of 50 Ω as required at the digital oscilloscope inputs. Baseband signals and demodulated constellations are displayed on Agilent Infiniium Digital Signal Oscilloscope, model 80804B.

The tested MMICs, or the improved ones in the future, can be used in a compact MHMIC/MMIC transceiver, fabricated in a mixed technology, as described in the previous section. Of course, the use of a more elaborated receiver allows a better signal to noise ratio of the receiving signals (the increased magnitudes of baseband signals) in addition to a demodulation rate of several Gb/s. For the current set-up, data rates of up to 1 Gb/s and a BER lower than 10^−8^ have been measured. This high data-rate is not important for a radar front-end, where baseband signals are in Hz to KHz range, but it can be critical in the future 5G/6G embedded automotive radar and ultra-high-speed communication systems.

This set-up also allows testing what is happening in the event of a frequency difference between the two multi-port millimeter wave inputs. This is very important in the case of a Doppler shift and, of course, for a radar sensor. As usual in a laboratory set-up, all signal generators are synchronized with a 10 MHz reference signal. By shifting, for example, the microwave frequency generator at LO port by 100 Hz, a 400 Hz frequency shift is obtained in V-band between the multi-port input frequencies.

**Figure 44:**
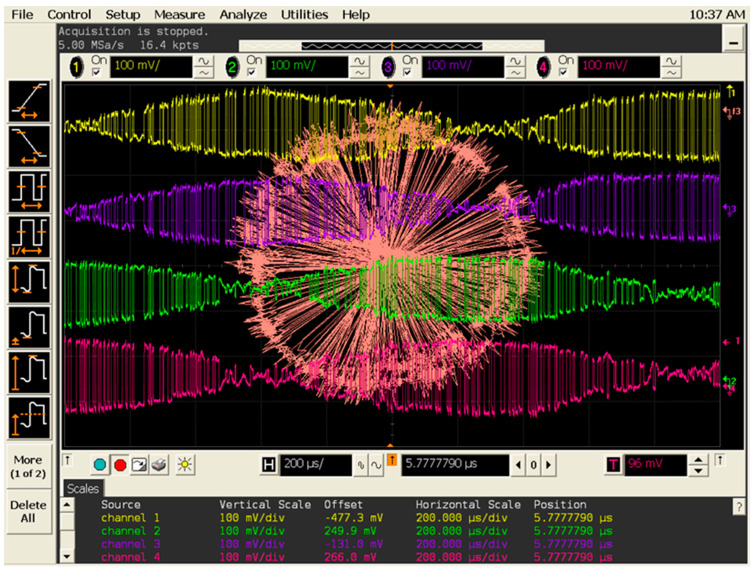
BPSK demodulated signals for a 400 Hz frequency shift between multi-port inputs.

Figure 44 shows a screenshot for a 250 KSa/s BPSK signal with an equivalent Doppler shift of 400 Hz. A modulation has been required because the transmitter does not work without any I/Q input voltages. BPSK modulation was chosen because it is easier to observe the phenomenon. Constellation points turn on a circle with the frequency of the Doppler shift. The period is 2500 µs, corresponding to 12.5 horizontal divisions. The quadrature differential signals at the multi-port detector outputs are always correctly phase opposite and phase shifted.

Quadrature demodulated signals passes successively by maximums and minimums, corresponding to the constellation rotation. Signal levels are in hundreds of mV range for the comparable distance between transmitter and receiver, as compared to those in mV ranges in the previous set-up (see waveforms in Figure 41) because LNA and baseband amplifiers are used. The voltage ratio of constellation diameters, as seen on the screens, is around 50 times bigger (34 dB), which is in accordance with the use of 20 dB LNA plus 20 dB baseband amplifier minus the gain of smaller dielectric lens.

It is to remember that antenna alignment is critical. Using the high gain cylindrical horn antennas and dielectric lenses, it is very easy the loss several dB for a fraction of one single degree of misalignment.

Frequency shifts from fractions of Hz (depending of the resolution of frequency synthesizers) to hundreds of MHz can be observed at the demodulator side. It was concluded that the multi-port is able to discriminate correctly the frequency difference, essential for wireless synchronization and speed measurements with radar sensors.

- Test-Bench #3

**Figure 45:**
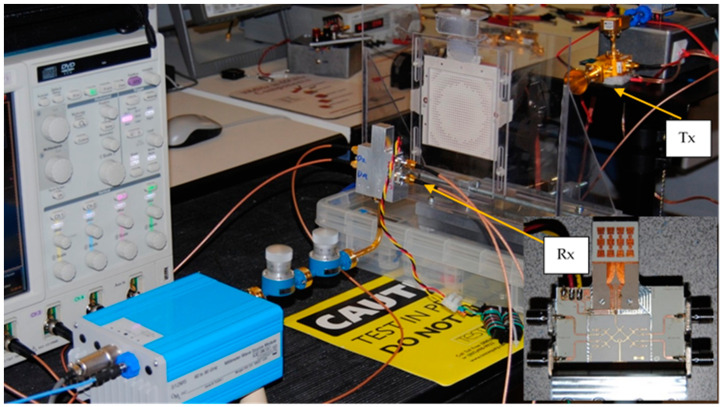
V-band multi-port receiver with integrated LNA and planar antenna array under test.

The V-band front-end with MMIC LNA of Figure 20 has also been tested using high-gain planar lens in a similar set-up as in the anechoic chamber, as seen in Figure 45. It is known from antenna measurements (see Figure 39b) that the antenna gain at the receiver is around 30 dBi at 61 GHz. A lower power transmitter (Tx) built with a passive V-band up-converter (−3 dBm output) is used in this set-up. The receiver (Rx) antenna system compensates the loss of the transmitted power versus the previous systems. Measurements show that constellations up to 32 symbols can be demodulated with a good S/N ratio. However, taking into account the huge band of the system (all ISM V-band), and the high-speed baseband amplifiers that are used, we notice that 2 Gb/s can be obtained even with a simple QPSK modulated signal. There is no need for more symbols for this value of data-rate.

#### 4.2.2. Multi-Port as Frequency Discriminator (IF and Low-IF Down-Converter)

- Test-Bench #4

The radar front-ends can also be tested in a simpler set-up, using for example as a source one of the millimeter wave extenders of the VNA. In our case, the VNA millimeter wave system includes the E 8362 PNA Network Analyzer and the N 5260A Millimeter Wave Head Controller from Keysight (Agilent) Technologies. Millimeter wave extenders, from OML Inc., optimized for Keysight (Agilent) N 5260A, are used for WR-12 waveguide S-parameter measurements.

**Figure 46:**
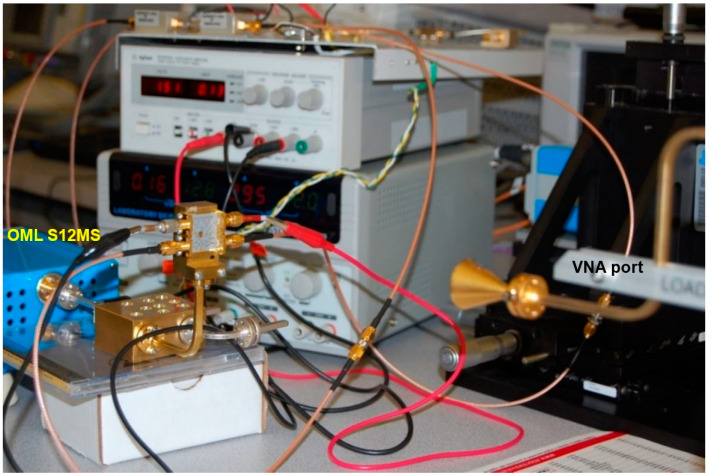
E-band multi-port receiver front-end under test with VNA source.

Figure 46 shows the radar measurement set-up, where the E-band radar front-end of Figure 16 is under test. The transmitting capability of the radar front-end was stopped using a matched load at the wave-guide coupler output. The multi-port is fed at the LO port by the same OML S12MS millimeter wave source module (6×), as in many of our previous set-ups. In order to generate a 77 GHz signal, the input microwave signal frequency is set at 13.833 GHz with 10 dBm power, required to drive the active multiplier.

The transmitter, as mentioned, uses a cylindrical horn antenna, the same as in the previous test-benches, connected to the millimeter wave extender. If different frequencies are set at the transmitter (VNA port) and at the receiver (LO), an IF signal is down-converted, according to the previous measurements and the multi-port theory.

**Figure 47:**
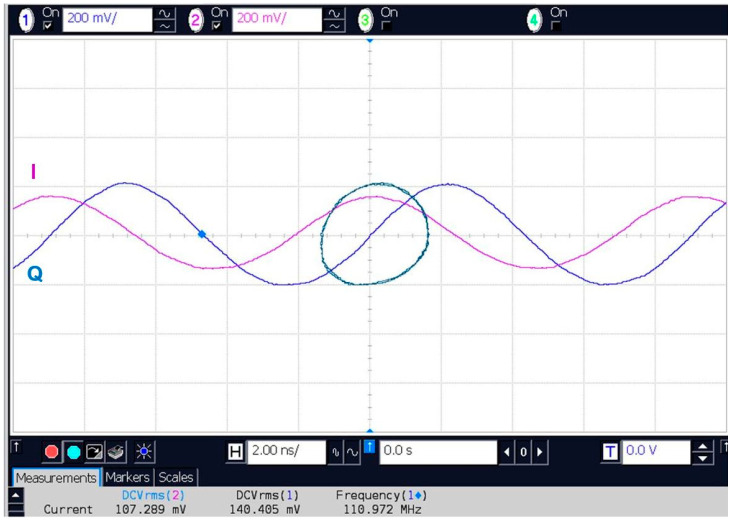
Quadrature baseband signals and the corresponding Lissajous graph.

The quadrature signals and the corresponding Lissajous graph are plotted in Figure 47. Results captured on the digital oscilloscope screen show I/Q IF signals for a frequency difference of around 111 MHz. As expected by the previous analysis of a multi-port in a communication system, this can be considered a Doppler shift. The Lissajous shape is close to a circular one. However, in typical radar applications, for a frequency discriminator, the aspect of this shape is not critical as long as it is a closed one. The number of turns/second is equivalent to the Doppler frequency, and this one is directly related to the target speed. In addition, for terrestrial targets, the Doppler shift is usually in KHz range. Because the front-end works up to several hundreds of MHz as a baseband (low IF) signal, it can also demodulate for example a FM, PSK, QAM signal, in a combined radar/communication system.

- Test-Bench #5

**Figure 48:**
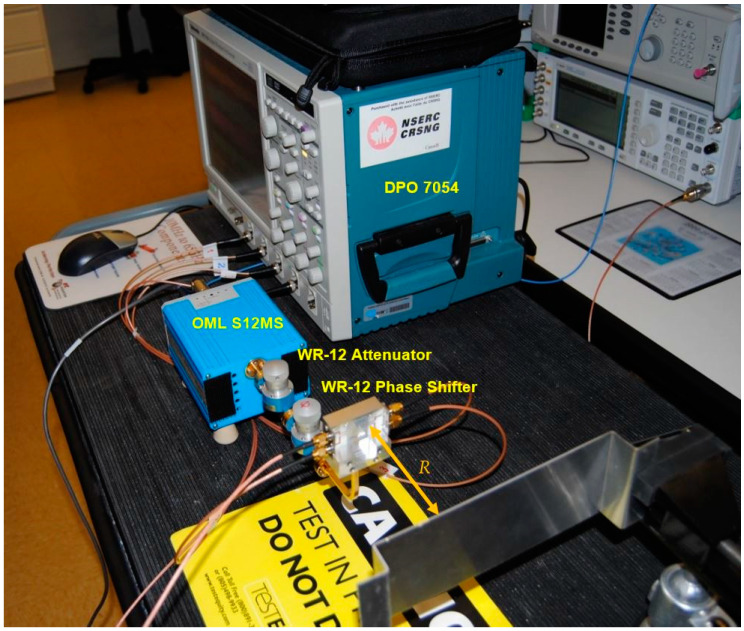
E-band radar in test with a metallic target.

Figure 48 shows the range measurement set-up using the radar sensor of Figure 9. This is a short-range sensor, because there are no LNA in reception or millimeter wave power amplifier in transmission. The microwave source frequency is set to 12.8333 GHz at 10 dBm power to feed the OML multiplier. This generates a 77 GHz signal with a power of around 7 dBm. At the input of the multi-port (on die, after WR-12 attenuator in series with the phase shifter and WR-12 to microstrip line transition part of the metallic fixture) we set the power at 0 dBm. A Tektronix DPO 7054 Digital Phosphor Oscilloscope is used to capture the detected output voltages of the radar sensor, that are subject to target movements.

It is known that for a radar system the received signal, after reflection on the target, is much smaller than in an equivalent communication system operating over the same range. The simplified radar equation is given by [95,96]:

P_Rx_ = P_Tx_ + G_Tx_ + G_Rx_ + 10log_10_{[σλ^2^]/[(4π)^3^R^4^]} (12)

In the previous radar equation for the received power P_Rx_ (dB), P_Tx_ is the power delivered to the terminals of an isotropic transmitting antenna (dB), G_Tx_ is the isotropic gain of the transmitting antenna (dBi), G_Rx_ is the isotropic gain of the receiving antenna, σ is the radar cross section of the target, and λ is the free space length and R is the range (distance to the target).

In our laboratory set-up of Figure 48, P_Tx_ = 0 dBm, G_Tx_ = G_Rx_ = 16 dBi, σ = 0.02 m^2^, and λ = 3.9 mm in free space at 77 GHz. The attenuation given by the last term of the Equation (12) is around −98 dB at R = 1 m, and −66 dB at R = 15 cm. According to the radar equation, the estimated received power at port 5 of the multi-port is therefore P_Rx_ = −66 dBm if the target is at R = 1 m and P_Rx_ = −34 dBm if the target is at R = 15 cm.

The estimated white noise level for the given bandwidth can be computed using the following equations [97,98]:

N = k T_0_ B (13)

N (dBm) = −174 + 10log_10_ (B) (14)

In the previous equations, k is the Boltzmann constant, k = 1.380649 × 10^−23^ J⋅K^−1^, T_0_ is the ambient temperature in °K, and B is the circuit bandwidth in Hz. For a 5 GHz bandwidth (76–81 GHz) and T_0_ = 290 °K (17 °C), the noise level is −77 dBm.

The target can therefore be set up to R = 1 m to have a received signal around 11 dB above the noise level with the current set-up, at the receiver input. At R = 15 cm the signal to noise ratio is more than 40 dB. These signal to noise values are also obtained at the radar receiver output, in the case of a perfect, non-internal noise receiver.

However, in real conditions, the noise figure F of the receiver, and the antenna temperature T_A_ must be taken into account. According to the theory of communication receivers, all receiver noise is transferred at its input, and the signal and the noise will be amplified with the same amount from its input to its output, for a constant signal to noise ratio [99].

The minimum detectable signal power level at the input is:

S_minRx_ = k B [T_A_ + (F − 1) T_0_](S_o_/N_o_) (15)

In the special case T_A_ = T_0_ this equation can be written as:

S_minRx_ = k T_0_ B F (S_o_/N_o_) (16)

S_minRx_ (dBm) = −174 + 10log_10_ (B) + F (dB) + S_o_/N_o_ (dB) (17)

Without any LNA, the noise figure of the front-end F is equal to its attenuation (passive circuit).

Using the measured S-parameters of the circuits, and inherent losses, we estimate that F = 10 dB. Considering also T_A_ = T_0_ = 290 K at the ambient temperature of 17 °C, according to (17), the noise floor level is raised by 10 dB, from −77 dBm, in the case of a perfect receiver, to around −67 dBm with a F = 10 dB of the receiver. Therefore, at R = 1 m the signal and noise level are comparable, no detection possible, for the actual transmitted power of only P_Tx_ = 0 dBm.

A minimum power signal level S_minRx_ = −57 dBm is obtained from (17) for the radar sensor if the signal to noise ratio S_o_/N_o_ = 10 dB is considered.

From (12) the relationship between the minimum required power level at the sensor input S_minRx_ (port 6 of the multi-port) and maximum detectable range R_max_ is:

S_minRx_ = P_Tx_ + G_Tx_ + G_Rx_ + 10log_10_{[σλ^2^]/ [(4π)^3^R_max_^4^]} (18)

The S_minRx_ (dBm) = −57 dBm, corresponds to a range of R_max_ = 59.1 cm, for actual P_Tx_ = 0 dBm. An increased transmitted power level with 10 dB to reach P_Tx_ = 10 dBm, using a millimeter wave power amplifier, will ensure P_Rx_ = −56 dBm, and therefore R = 1 m is in the range of the radar sensor.

Range (R) measurement demonstration is performed in two steps on the test-bench [68]. First, the target is set at a known distance. Second, by using the information from the sensor, the distance is calculated and compared to the known distance. Furthermore, using only the information from the sensor, other distances can be measured.

**Figure 49:**
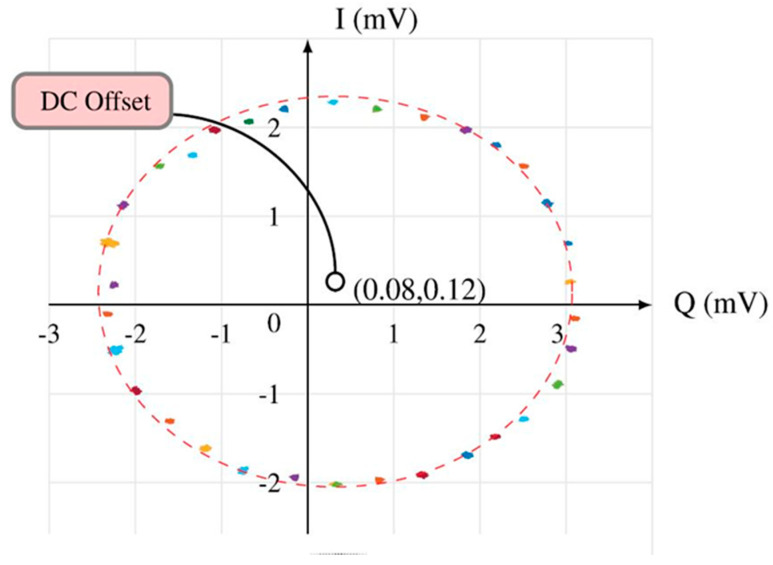
I/Q points over 1 GHz frequency shift.

The target (an aluminum plate with a radar cross section σ = 0.02 m^2^) is first placed R = 15 cm from the fabricated sensor, and the phase shifts are measured for various frequencies in steps of ∆ f = 30 MHz from 76.5 GHz to 77.5 GHz, as seen in Figure 49. In fact, instead of physically changing the range R, the electrical length and the number of wavelengths were changed accordingly by changing the LO frequency. It would be difficult to make a calibrated moving mechanical target as accurate. Both approaches to experimentally validate the model are equivalent. Expected phase shift for each step is given by the following equation [59,68]:

∆ ϕ_2_ − ∆ ϕ_1_ = 4πR (f_2_ − f_1_)/c (19)

As expected, all points move around a circle with a specific phase shift of 10.8°, which agrees with the target position and expected target range obtained from Equation (17).

A complete tour of 360° in I/Q complex plane is obtained for 1 GHz frequency difference of the LO, and the range is calculated such as:

R = (∆ ϕ_2_ − ∆ ϕ_1_) c / 4π (f_2_ − f_1_) = 2πc/4π (f_2_ − f_1_) = 15 cm (20)

The center of the circle represents the DC-offset of the circuit, which depends on the characteristics of the multi (six)-port circuit, on the differences between the four power detectors at the output ports, and on the settings of the oscilloscope if DC coupling is used. The radar front-end can operate over a 5 GHz band, from 76 to 81 GHz, allowed for radar sensors, f_2_ − f_1_ = 5 GHz. Considering a ∆ ϕ resolution of 5°, easily to measure, we can obtain a radar resolution of 0.41 mm from Equation (20).

- Test-Bench #6

Tuning fork experiment is performed with the same radar sensor front-end. A tuning fork is placed in front of the sensor. This specific moving target mechanical vibrations modulate the carrier RF signal in a non-linear way [41]. As observed in the previous experiment, small movements of the target modulate the phase of the reflected signal. If the mechanical vibration is less than λ/2 phase variations are less than 360°. In this project, tuning forks are used as targets, which can produce exponentially-damped sinusoidal movements. Therefore, to simplify the analysis, and without any loss of generality, we consider the sinusoidal movement to be in the steady-state response of the fork movement.

As discussed in [67,100,101], the baseband signal at the output of the sensor can be expressed as a Bessel function expansion of order n, Jn(x). For a single tone target movement, x(t) = m sin (2π f t), the baseband output signal can be expressed as:

(21) $$B\left(t\right)=\overset{\infty }{\underset{n=-\infty }{\sum }}J_{n}\left(\frac{4\pi m}{\lambda }\right)\cos\left(2\pi \mathrm{nf}\right)\cos\left(\frac{4\pi R}{\lambda }\right)$$

Equation (21) shows that harmonics will be created in baseband output signal due to the non-linear property of the cosine transfer function. The tuning fork fundamental frequency is obtained from the fundamental frequency of B (t). The harmonics generated by the non-linear phase modulation can be used to recover the desired information about movement of the target [67,102].

**Figure 50:**
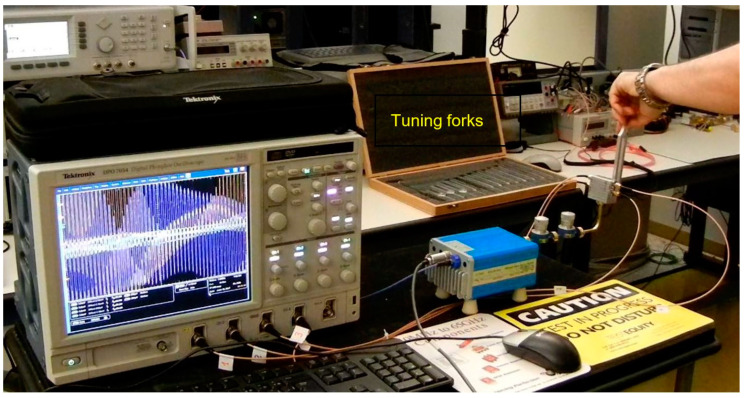
E-band radar sensor test-bench for tuning fork experiment.

Figure 50 shows a photo of the test bench. It is similar with the one described in Figure 48, with the exception of the target, which is now a tuning fork. For larger forks, having a lower oscillation frequency, important harmonic contributions are observed in the beginning due to the higher amplitude of mechanical oscillations, as seen on the oscilloscope screen.

**Figure 51:**
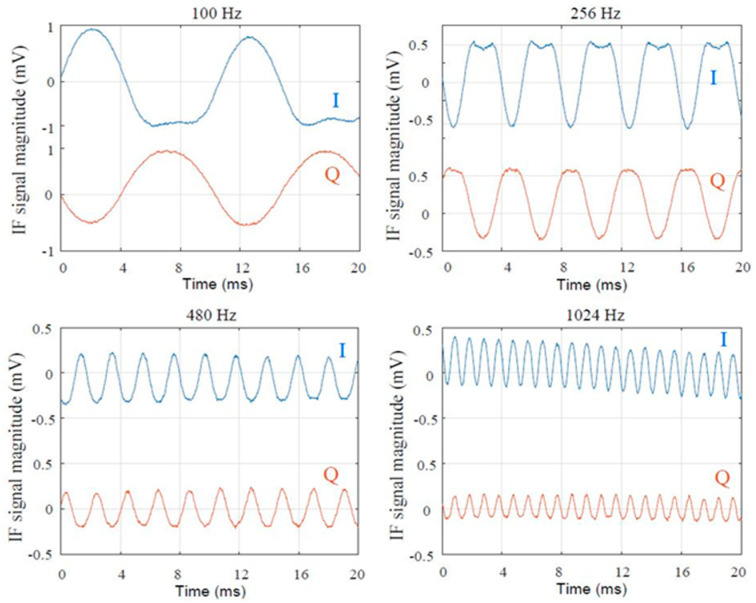
Measurement results of quadrature signals for different tuning forks.

Tuning forks from 100 Hz to 4096 Hz have been tested. Waveforms of some quadrature signals are shown in Figure 51 [67].

For lower audio frequencies the total oscillation time (from the beginning when the fork is exited with the rubber hammer until the listening of a pure tone which slowly disappeared) is longer, due to the higher amplitude vibration of the fork. At the end of the mechanical oscillations, when harmonics are close to zero compared to the fundamental, the audio frequency of the calibrated tuning fork, with a +/−1-Hz precision, can be measured on the screen using the measurement tools of the digital oscilloscope. The amplitude of the signals corresponding to lower audio frequency is of course bigger, for the same reasons, as also seen in the previous figure.

This experiment demonstrates the accuracy of the mechanical frequency measurement using the radar sensor, which is in the limits of the precision of the state-of-the-art digital oscilloscopes.

- Test-Bench #7

A metronome experiment is also performed using the same radar sensor front-end. These measurements intend to validate that the sensor can read with accuracy even lower frequencies than in the previous case of the tuning fork experiment. A NIKKO standard mechanical metronome, having 40 to 208 oscillations/minute (0.666–3.466 Hz) has been used as moving target.

**Figure 52:**
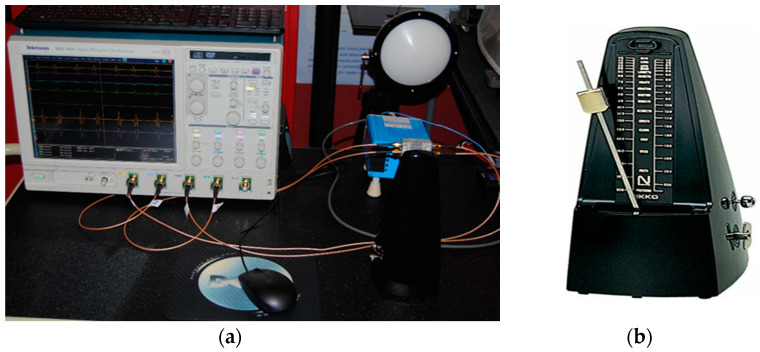
E-band radar sensor test-bench for metronome experiment: (**a**) overall set-up; (**b**) metronome.

Figure 52a shows the test-bench and the details on the moving target (metronome pendulum) can be seen in the metronome picture of Figure 52b. The amplitude of oscillation of the baseband quadrature signals does not exceed several mV because of the very small radar cross section and high free space attenuation losses. However, the sensor clearly identifies the passes of the target in front of the antennas.

The sensor can be therefore used to measure vital signs (heartbeat or respiration rate) wirelessly. Gain enhancement, both at millimeter wave and baseband frequencies, will dramatically improve the measurement range, from several cm (without any amplifiers) to dozens of cm or even several meters.

## 5. Discussion

Fabrication and characterization techniques of millimeter wave circuits and front-end modules described in the paper are very useful today, in the novel context of widespread usage of these frequency bands, especially in prototyping or small-scale production.

The millimeter wave interferometry, a simple and efficient quadrature down-conversion technique, is used in this paper as a suggestive example in the design of front-ends for radar applications. Basically, for radar or communication systems, the interferometric front-end design is very similar. It allows the use of the same module in combined communication/radar transceivers for example for the 5 G and beyond automotive applications (autonomous vehicles, vehicle-to-everything communications, etc.).

Measurement results show the accuracy of the frequency and phase measurements for a RF modulated signal or a mechanical oscillation. In fact, precision is limited basically by the measurement equipment connected to the radar sensor quadrature differential baseband outputs. Frequency measurements are performed from fractions of Hz until hundreds of MHz range. Phase accuracy is in the range of several degrees, basically limited by the inherent noise. The measurement system does not require any calibration for frequency measurements, it only requires DC offset compensation for phase measurements within an error of several degrees. For more accurate phase measurements, a simple calibration procedure is recommended, to transform the elliptical Lissajous shape of the XY display into a circular one.

The size of millimeter wave front-ends is basically limited by the antenna array surface, which is directly related to the free space wavelength and the required gain. Therefore, circuit miniaturization reaches a physical limit.

The use of the proposed hybrid fabrication techniques, with integrated MMIC chips embedded on the MHMIC planar surface, allows a wide opening in system architecture prototyping and testing, before medium and large-scale production.

Future research will be done in (i) design of improved front-ends with state-of-the art MMIC, (ii) design of in-house MMICs targeting better performances than commercial ones for a specific application and (iii) efficient millimeter wave (analog) and baseband (digital) signal processing. These activities will be related for each specific targeted application: automotive, industrial, medicine (such as vital sign detection), gesture sensing for human to machine communication, etc.
